# Comparative proteomics illustrates the complexity of drought resistance mechanisms in two wheat (*Triticum aestivum* L.) cultivars under dehydration and rehydration

**DOI:** 10.1186/s12870-016-0871-8

**Published:** 2016-08-31

**Authors:** Lixiang Cheng, Yuping Wang, Qiang He, Huijun Li, Xiaojing Zhang, Feng Zhang

**Affiliations:** 1 College of Agronomy, Gansu Provincial Key Laboratory of Aridland Crop Science, Gansu Key Laboratory of Crop Improvement & Germplasm Enhancement, Research & Testing Center, Gansu Agricultural University, Lanzhou, China; 2 Wuwei Agricultural and Animal Husbandry Bureau, Wuwei, China; 3 Gansu Dingxi Academy of Agricultural Science, Dingxi, China

**Keywords:** Wheat, Drought stress, Differentially abundant proteins, Proteomics, 2-DE, MALDI-TOF/TOF

## Abstract

**Background:**

Drought stress is one of the most adverse environmental constraints to plant growth and productivity. Comparative proteomics of drought-tolerant and sensitive wheat genotypes is a strategy to understand the complexity of molecular mechanism of wheat in response to drought. This study attempted to extend findings regarding the potential proteomic dynamics in wheat under drought stress and to enrich the research content of drought tolerance mechanism.

**Results:**

A comparative proteomics approach was applied to analyze proteome change of Xihan No. 2 (a drought-tolerant cultivar) and Longchun 23 (a drought-sensitive cultivar) subjected to a range of dehydration treatments (18 h, 24 h and 48 h) and rehydration treatment (R24 h) using 2-DE, respectively. Quantitative image analysis showed a total of 172 protein spots in Xihan No. 2 and 215 spots from Longchun 23 with their abundance significantly altered (*p* < 0.05) more than 2.5-fold. Out of these spots, a total of 84 and 64 differentially abundant proteins were identified by MALDI-TOF/TOF MS in Xihan No. 2 and Longchun 23, respectively. Most of these identified proteins were involved in metabolism, photosynthesis, defence and protein translation/processing/degradation in both two cultivars. In addition, the proteins involved in redox homeostasis, energy, transcription, cellular structure, signalling and transport were also identified. Furthermore, the comparative analysis of drought-responsive proteome allowed for the general elucidation of the major mechanisms associated with differential responses to drought of both two cultivars. These cellular processes work more cooperatively to re-establish homeostasis in Xihan No. 2 than Longchun 23. The resistance mechanisms of Xihan No. 2 mainly included changes in the metabolism of carbohydrates and amino acids as well as in the activation of more antioxidation and defense systems and in the levels of proteins involved in ATP synthesis and protein degradation/refolding.

**Conclusions:**

This study revealed that the levels of a number of proteins involved in various cellular processes were affected by drought stress in two wheat cultivars with different drought tolerance. The results showed that there exist specific responses to drought in Xihan No. 2 and Longchun 23. The proposed hypothetical model would explain the interaction of these identified proteins that are associated with drought-responses in two cultivars, and help in developing strategies to improve drought tolerance in wheat.

**Electronic supplementary material:**

The online version of this article (doi:10.1186/s12870-016-0871-8) contains supplementary material, which is available to authorized users.

## Background

Drought is one of the most adverse environment stress factors that negatively affects plant growth and development, which adversely leads to the yield losses of major crops worldwide every year [1]. Of the 1.5 billion hectares of global cropland, only 20 % were irrigated that provides about 40 % of the world’s food production, whereas the remaining 60 % was provided by rain-fed agriculture. Wheat (*Triticum aestivum* L.) as the world’s most important cereal crop is grown in a large range of latitudes worldwide under both irrigated and rain-fed conditions and thus in conditions subjected to drought. Wheat is considered as an excellent system to study drought tolerance in spite of its genetic complexity [2]. Recently, the completion of chromosome-based draft sequencing of hexaploid bread wheat genome will accelerate wheat breeding and identification of key genes controlling complex traits in response to drought [3]. Based on the wheat genome sequencing data, much research effort would be applied to gain better understanding of mechanisms adopted by wheat to combat drought stress.

To date, some physiological and molecular mechanisms of plants to cope with drought stress have been extensively described by many researchers. When plants are subjected to drought stress, an early response is the rapid closure of stomata triggered by ABA for decreasing water loss from leaves [4, 5]. The transient increases of ABA level under water deficit condition can also trigger many downstream stress responses [6]. Two major responses have emerged in terms of cellular and molecular basis in coping with drought. First, the initial response is closely related to osmotic adjustment [7]. The accumulated osmolytes include proline, glutamate, glycine-betaine and sugars (mannitol, sorbitol and trehalose), which play a key role in preventing membrane disintegration and enzyme inactivation under drought stress [8, 9]. Second, a large number of drought-responsive genes and specific protective proteins were induced for drought tolerance [10, 11]. These drought stress-related transcripts and proteins are mostly involved in signalling transduction and activation/regulation of transcription, antioxidants and reactive oxygen species (ROS) scavengers [12]. Most of the important transcription regulon in drought-induced ABA signalling pathways have been identified, such as dehydration-responsive element binding protein (DREB), C-repeat-binding factor (CBF), ABA-responsive element binding protein (AREB), ABA-binding factor (ABF), myelocytomatosis oncogene (MYC) and myeloblastosis oncogene (MYB) [13–15]. DREB and CBF function in ABA-independent gene expression, whereas AREB, ABF, MYC and MYB function in ABA-dependent gene expression [16]. In wheat, stress-inducible expression of *TaDREB2* and *TaDREB3* genes demonstrated substantial resistance to drought stress [17]. Over-expression of *TaNAC69* leads to enhanced transcript levels of stress up-regulated genes and dehydration tolerance in bread wheat [18]. A MYB gene from wheat, *TaMYBsdu1*, is up-regulated under drought stress and differentially regulated between tolerant and sensitive genotypes [19]. For the ROS-scavenging pathways, the deleterious effects of ROS under drought stress need to be quickly scavenged to protect cells from oxidative damage. Some antioxidant enzymes, such as superoxide dismutase (SOD), catalase (CAT), ascorbate peroxidase (APX), glutathione peroxidase (GPX), glutathione reductase (GR) and glutathione S-transferase (GST), are responsible for ROS-scavenging [6]. Drought-induced up-regulation of these proteins suggested the presence of well-equipped antioxidant system in plant cells to cope with drought stress [20, 21]. Apart from antioxidants, accumulation of molecular chaperons (HSP17, HSP70, Chap60, dnaK) helps in refolding of misfolded proteins [22]. In addition, inducible synthesis of dehydrin (DHN) proteins further provides protection to membranes against dehydration damage [23]. The association between accumulation of *DHN* family members and drought tolerance has been shown in some species, such as wheat [24, 25], tomato [26], gentian [27] and white clover [28].

Despite intensive studies on drought-responsive mechanisms in plants [29–32], drought tolerance mechanisms remain largely unknown due to a complex nature of the quantitative trait. It is known that different cultivars within a crop species may greatly differ in their response and adaptation to drought stress [21, 33]. The information available on the molecular basis of drought tolerance in different wheat genotypes is still limited. Previous studies at transcriptomic level have revealed that the drought-tolerant and sensitive wheat genotypes can adopt different molecular strategies to cope with drought stress [34–37]. One of the main differences is the differential expression of some drought-inducible genes for protection (e.g., antioxidants, detoxifiers, dehydrins, transporters and compatible solutes), regulation (e.g., kinases, transcription factors and hormones) and remodelling (e.g., membrane systems, cell wall and primary metabolic networks) [25, 30, 31, 37]. A large number of these well-known drought-related genes can often be activated in drought-sensitive wheat genotype, while the tolerant genotype shows the constitutive expression of several genes activated by drought in sensitive genotype, which might contribute to limit drought effect and perception [37]. In addition, signal transduction and hormone-dependent regulation pathways are also different in different wheat genotypes [35, 38]. The drought-tolerant genotype can quickly sense drought and trigger the signal transduction pathways for activation of downstream elements for survival from drought stress. The differential expression of *phospholipase C* gene involved in inositol-1, 4, 5-triphosphate (IP_3_) signalling and mitogen-activated protein kinase (MAPK) cascade elements has been reported in two contrasting wheat genotypes [35]. Some transcription factors also have unique responses to drought stress in different wheat genotypes, suggesting differences in hormone-dependent regulation pathways. A drought-tolerant wheat genotype has been reported to show induction of *bZIP* and *HD-ZIP* gene known as transcription factors relevant to ABA regulatory pathway under drought stress, whereas the sensitive genotype induced transcription factors that bind to ethylene responsive elements [35]. Although these studies have provided important insights to some extent, the data at transcriptional level are always insufficient to predict protein expression due to post-transcriptional and post-translational regulation mechanisms [39]. There is far less information available on the functional products of these identified genes, leading to poor knowledge of correlations between transcriptomes and proteomes in drought-tolerant and sensitive wheat genotypes under drought stress.

Proteomics has become the most direct and powerful tool to obtain protein expression information of plants in response to drought stress [9, 20]. It can provide the global protein expression profile encoded by genome, thereby complementing transcriptomic studies [40]. Comparative proteomics of drought-tolerant and sensitive wheat genotypes is a strategy to understand the complexity of molecular mechanism of wheat in response to drought stress. Recently, a few published studies have been applied to describe proteome changes in different wheat genotypes under drought stress [41–45]. A small set of drought-inducible proteins was also identified from these studies in various wheat organs including seedling leaves, stems, roots and grains. Differential expression of these proteins in different wheat genotypes may be responsible for the stronger drought resistance of tolerant genotypes. Although these studies have provided some insight into the molecular mechanisms of different wheat genotypes in response to drought stress, the limited information cannot be enough to establish the possible drought-responsive proteins network for explaining the different drought-responsive strategy in drought-tolerant and sensitive genotypes. Furthermore, it is conceivable that there may be many novel drought-inducible proteins yet to be identified in previous studies. Thus, our observations attempt to extend findings regarding the potential proteomic dynamics in drought-tolerant and sensitive wheat genotypes under drought stress and to enrich the research content of drought tolerance mechanism.

In the present study, a comparative proteomics approach was applied to investigate the molecular events of two wheat cultivars in response to drought stress, Xihan No. 2 (drought-tolerant cultivar) and Longchun 23 (drought-sensitive cultivar), respectively. The differentially abundant proteins including well-known and novel drought-responsive proteins were identified in two cultivars under drought stress using 2-DE coupled with MALDI-TOF/TOF MS and Mascot database searching. The findings will help drive further work to develop strategies for improving drought tolerance and water use efficiency of wheat, and to gain comprehensive knowledge of the underlying molecular mechanisms involved in drought response.

## Methods

### Plant materials, growth conditions and dehydration treatments

Seeds of wheat (*Triticum aestivum* L. cvs. Xihan No. 2 and Longchun 23) were supplied by Gansu Provincial Key Laboratory of Aridland Crop Science, Lanzhou, China. The two wheat cultivars were different in drought resistance. In arid area with a rainfall of 250–300 mm, the average yield of Xihan No. 2 (a drought-tolerant cultivar, approved by the National Crop Variety Approval Committee of China in 2007) was 15–45 % higher than Longchun 23 (a drought-sensitive cultivar), which itself produced only 50 % of the yield compared with optimal watering. The seeds of two cultivars were sucking water to break seed dormancy for 2 days at 25 ± 2 °C, then they were sown in glass plates containing expanded perlite in an environmentally controlled growth room with 25 ± 2 °C, 70 % relative humidity and 16 h photoperiod (300 μmol m^−2^ · s^−1^ light intensity). Initially, the plants were irrigated with 300 ml of water every day that maintained the moisture content at about 30 %. After a week, drought treatment was carried out in 1-week-old seedlings by withholding water for 48 h, and then re-watered for the recovery of dehydrated seedlings. The leaf samples were taken in triplicate from both stressed/re-watered plants and continuously watered controls after 18 h, 24 h and 48 h of dehydration and 24 h of rehydration, respectively. The samples from controls were collected at each time point during dehydration and were finally pooled to normalize the growth and developmental effects. The fresh leaves were directly used to determine the physiological and biochemical responses of wheat seedlings under drought stress. Another part of leaves was immediately frozen in liquid nitrogen and stored at −80 °C until the further processing of proteomic analysis.

### Determination of relative water content

The relative water content (RWC) was measured as described by Bhushan et al. [9]. Fresh leaves were sampled and immediately weighted for fresh weight (FW). To determine turgid weight (TW), the leaves were incubated in distilled water in darkness at 4 °C for 24 h to minimize respiration losses until fully turgid. Dry weight (DW) was determined by drying the fully turgid leaves in an oven at 80 °C for 48 h. The RWC was calculated by the following formula: RWC (%) = [(FW - DW) / (TW - DW)] × 100.

### Determination of proline accumulation

Proline was extracted and determined by the method of Bates et al. [46]. Approximately 0.5 g of fresh leaves was homogenized in 5 ml of 3 % (w/v) aqueous sulfosalicylic acid. The homogenate was centrifuged at 5 000 × g for 15 min at 4 °C. The supernatant was treated with acid ninhydrin reagent and glacial acetic acid (1:1, v/v), boiled at 100 °C for 1 h, then the reaction was terminated on ice for 5 min. The absorbance of reaction mixture was read at 520 nm. Proline content was determined from standard curve and calculated on a fresh weight basis (μg · g FW^−1^).

### Determination of lipid peroxidation

Malonaldehyde (MDA) content as an important index of lipid peroxidation was measured following the methods of Hodges et al. [47]. Approximately 0.5 g of fresh leaves was homogenized in 5 ml of 0.1 % (w/v) trichloroacetic acid (TCA). The homogenate was centrifuged at 10 000 × g for 15 min at 4 °C, and 1 ml of supernatant was added to 2 ml of 0.5 % (v/v) TBA in 20 % TCA. The mixture was incubated at 100 °C for 30 min and then quickly cooled in an ice bath. After centrifuged at 10 000 × g for 10 min at 4 °C, the absorbance of supernatant was recorded at 450 nm, 532 nm and 600 nm, respectively. The non-specific absorbance at 600 nm was subtracted, and a standard curve of sucrose was used to rectify the possible interference of soluble sugars in samples. MDA content was calculated using an extinction coefficient of 155 mM^−1^ cm^−1^and expressed as μg · g FW^−1^.

### Determination of electrolyte leakage

Electkrolyte leakage was assayed according to Yan et al. [48]. Fresh leaves were cut into 1 cm segments and washed three times with ultrapure water. The segments were incubated in a tube containing 10 ml of ultrapure water at room temperature for 2 h. Two hours later, conductivity (C_1_) was recorded using a conductivity meter (INESA, China). Then, the tubes were incubated at 100 °C for 20 min. After the solution was cooled to room temperature, conductivity (C_2_) was recorded again. Electrolyte leakage was calculated by the following formula: Electrolyte leakage (%) = C_1_ / C_2_ × 100.

### Determination of photosynthetic pigments

Approximately 1 g of fresh leaves was extracted in 10 ml of 80 % chilled acetone. After centrifuged at 3 000 × g for 2 min at 4 °C, the supernatant was used for the determination of photosynthetic pigments. The absorbance of supernatant was recorded at 663 nm, 645 nm and 470 nm, respectively. Chlorophyll and carotenoid content was calculated as described by Bhushan et al. [9] and expressed as mg · g FW^−1^.

### Determination of H_2_O_2_ content

H_2_O_2_ content was determined by the peroxidase-coupled assay according to Veljovic-Jovanovic et al. [49]. Approximately 0.2 g of fresh leaves was ground in liquid nitrogen and the powder was extracted in 2 ml of 1 M HClO_4_ in the presence of 5 % insoluble polyvinylpyrrolidone (PVP). The homogenate was centrifuged at 12 000 × g for 10 min and the supernatant was neutralized with 5 M K_2_CO_3_ to pH 5.6 in the presence of 100 ml 0.3 M phosphate buffer (pH 5.6). The solution was centrifuged at 12 000 × g for 1 min and the sample was incubated for 10 min with 1 U ascorbate oxidase (Sigma, St. Louis, USA) to oxidize ascorbate prior to assay. The reaction mixture consisted of 0.1 M phosphate buffer (pH 6.5), 3.3 mM DMAB (3-dimethylaminobenzoic acid) (Sigma, St. Louis, USA), 0.07 mM MBTH (3-methyl, 2-benzo thiazolinone hydrazone) (Sigma, St. Louis, USA) and 0.3 U POX (peroxidase) (Sigma, St. Louis, USA). The reaction was initiated by addition of 200 ml sample. The absorbance change at 590 nm was monitored at 25 °C.

### Enzyme assay

Approximately 1 g of fresh leaves was homogenized in 5 ml of extraction buffer [50 mM K-phosphate buffer (pH 7.8), 1 mM Na-EDTA and 1 % (w/v) PVP]. The homogenate was centrifuged at 15 000 × g for 20 min at 4 °C, and the supernatant was used to assay the enzyme activity. All the steps in the preparation of enzyme extracts were performed at 4 °C. Total superoxide dismutase (SOD) activity was measured by nitroblue tetrazolium (NBT) method of Beyer & Fridovich [50] and expressed as units · mg protein^−1^. Catalase (CAT) activity was assayed by monitoring the consumption of H_2_O_2_ at 240 nm (E = 39.4 mM^−1^ cm^−1^) according to the method of Aebi [51] and expressed as μmol · min^−1^ · mg protein^−1^.

### Protein extraction

Total leaf proteins were extracted from the control and treatment seedlings as described by Donnelly et al. [52] with some modifications. Approximately 2 g of leaves were homogenized in liquid nitrogen. The homogenate was precipitated overnight at −20 °C by the addition of 25 ml of chilled 10 % (w/v) TCA/acetone containing 1 mM PMSF and 0.07 % (v/v) β-mercaptoethanol. After centrifuged at 20 000 × g for 20 min at 4 °C, the pellet was collected and incubated at −20 °C for 20 min. Then pellet was washed and resuspended with 20 ml of chilled acetone containing 1 mM PMSF and 0.07 % (v/v) β-mercaptoethanol. After centrifuged at 15 000 × g for 15 min at 4 °C, the pellet was collected and incubated at −20 °C for 10 min. The steps were repeated until the pellet became pure white. The washed pellet was air-dried for 1 h and then solubilized in 250 μl of rehydration buffer [8 M urea, 2 % (v/v) Triton X-100, 1 % (w/v) DTT, 1 mM PMSF] for 2 h at room temperature. After centrifuged at 15 000 × g for 15 min at 4 °C, the supernatant was collected and stored at −80 °C. The protein extraction was repeated three times, and the protein concentration was measured using Bio-Rad Protein Assay Kit (Bio-Rad, Hercules, CA, USA) according to the manufacturer’s instructions with bovine serum albumin (BSA) as the standard.

### 2-DE (Two-dimensional polyacrylamide gel electrophoresis)

The first dimension of the isoelectric focusing (IEF) was performed using 17 cm immobilised pH gradients (IPG) strips (Bio-Rad, Hercules, CA, USA) with pH gradients 3–10 in PROTEAN IEF Cell System (Bio-Rad, Hercules, CA, USA). The IPG strips were rehydrated overnight with 900 μg of total proteins diluted in rehydration buffer [7 M urea, 2 M thiourea, 2 % (w/v) CHAPS, 0.3 % (w/v) DTT, 0.5 % (v/v) IPG buffer (pH3-10) and 0.001 % (w/v) bromophenol blue] to reach a final volume of 350 μl. After rehydration, the focusing was performed at 20 °C using the following settings: 50 V during 14 h, gradient to 250 V during 0.5 h, gradient to 1 000 V in 1 h, gradient to 10 000 V in 5 h, 10 000 V until 60 000 Vh. Prior to second dimension electrophoresis, the IPG strips were equilibrated at room temperature for 15 min in 5 ml of equilibration buffer [6 M urea, 2 % (w/v) SDS, 20 % (v/v) glycerol, 0.375 M Tris-HCl (pH8.8) and 0.2 % (w/v) DTT], and subsequently for 15 min in the same buffer but 2.5 % (w/v) iodoacetamide replacing DTT. The equilibrated strips were loaded and run on 12 % SDS-PAGE gels using PROTEANII xi Cell System (Bio-Rad, Hercules, CA, USA) with a programmable power controller. The gels were run for 15 min at 50 V, then at constant voltage 200 V until the dye front reached the bottom of gel. The separated proteins were visualized by coomassie brilliant blue (CBB) G-250 staining. For each protein sample, three replicates were run for each gel to ascertain reproducibility.

### Image acquisition and data analysis

The CBB-stained 2-DE gels were scanned with a UMAX PowerLook 2100XL-USB scanner (Maxium Tech Inc., Taiwan, China) at 600 bits per pixel and scan resolution of 300 dpi in a transmission mode. Image analysis was subsequently carried out with PDQuest v8.0.1 software (Bio-Rad, Hercules, CA, USA), including background subtraction, spot detection, spot measurement and spot matching. The gel image of control was selected as a reference gel to align with gel image of dehydration (18 h, 24 h and 48 h) and rehydration (R24 h), respectively. The abundance of one protein spot was expressed as the volume of that spot which was defined as the sum of the intensities of all the pixels that make up that spot. To minimize possible errors due to differences in the amount of protein loaded and the staining intensity, the spot abundance was normalised as a percentage of the total spot volume in the gel. The normalised percentage volume (Relative V%) of protein spots from triplicate biological samples were subjected to statistical analysis using means ± standard error (SE). At least nine images derived from three biological replicates of each treatment were compared, which were obtained in the same experimental set. We used one-way analyses of variance (ANOVA) to evaluate the significance (*p* < 0.05) of protein differential expression. Only spots with statistical significance (*p* < 0.05) and reproducible changes were considered, and the spots with an abundance ratio at least 2.5-fold in relative abundance were selected as differentially abundant proteins. These spots were then selected for protein identification using MALDI-TOF/TOF MS.

### Tryptic digestion

Spots with significantly differential expression from 2-DE gels were carefully excised. Gel spots were washed twice for 30 min with deionized water, and then destained and dehydrated with acetonitrile (ACN). After washed twice for 30 min at room temperature with vigorous shaking in 400 μl of 50 % ACN containing 50 mM ammonium bicarbonate, the gel spots were incubated overnight with 400 μl of 100 % ACN and then dried. Proteins were digested for 18 h at 37 °C in 10 μl of 15 ng/μl trypsin solution. The supernatant was collected, and the fluid was further extracted twice from gel spots with 50 μl of 50 % ACN containing 5 % trifluoroacetic acid (TFA) for 1 h at 37 °C. Finally, all the extractions were pooled with the trypsin supernatant and dried.

### Protein identification by MALDI-TOF/TOF MS

For MALDI-TOF/TOF MS, digested protein samples were mixed (1:1, v/v) with the matrix solution [7 mg/ml α-cyano-4-hydroxycinnamic-acid in 50 % (v/v) ACN and 0.1 % (w/v) TFA], and then 0.7 μl of this mixture was spotted on the MALDI target. Tryptic peptides were analysed using an ABI 4800 Plus MALDI-TOF/TOF™ Analyzer (AB SCIEX, Framingham, MA, USA). The MS spectra were recorded in the positive reflector mode in a mass range from 800 to 4000 with a focus mass of 2000. For one main MS spectrum 25 subspectra with 125 shots per subspectrum were accumulated using a random search pattern. MS was used a CalMix5 standard to calibrate the instrument (ABI 4700 Calibration Mixture). For MS calibration, autolysis peaks of trypsin (m/z 842.5100 and 2211.1046) were used as internal calibrates, and up to 10 of the most intense ion signals were selected as precursors for MS/MS acquisition, excluding the trypsin autolysis peaks and the matrix ion signals. In MS/MS positive-ion mode, for one main MS spectrum 50 subspectra with 50 shots per subspectrum were accumulated using a random search pattern. Collision energy was 1-kV, collision gas was air, and default calibration was set by using the Glu1-Fibrino-peptide B (m/z 1570.6696) spotted onto Cal 7 positions of the MALDI target. Both the MS and MS/MS data were integrated and extracted using GPS Explore v3.6 software (AB SCIEX, Framingham, MA, USA). Peptides were identified by searching for taxonomy (*Viridiplantae*, green plants; 1022713 sequences) in the NCBInr database 20120107 (16831682 sequences; 5781564572 residues) using Mascot v2.2 search engine (Matrix science, London, UK). The parameters for searching were: enzyme equals trypsin; one missed cleavage; allowed variable oxidation modifications (Met); allowed fixed modifications of carbamidomethyl (Cys); peptide mass tolerance of 100 ppm; fragment mass tolerance of 0.3 Da. The significance threshold (*p* < 0.05) was set using the Mascot algorithm.

### Functional classification and hierarchical clustering analysis

The functional classification of the identified proteins was conducted according to the putative functions assigned to each of the candidates using the protein function database. A hierarchical clustering analysis was performed by using the Multi Experiment Viewer (MEV) software (Pearson correlation, default parameters). The data were taken in terms of -fold expression with respect to the control expression value. Then, the data sets were log-transformed to the base 2 to level the scale of expression and reduce the noise. Only the clusters with *n* > 6 were taken to investigate the co-expression patterns for functionally similar proteins.

### Statistical analysis

Statistical analysis was carried out with three biological replicates for proteomic and physiological analyses. The repeated measurement was given as means ± standard error (SE). The results of spot abundance and physiological data were statistically evaluated by one-way analyses of variance (ANOVA) and the Duncan’s multiple range test to determine the significant difference among group means. In all cases, significance was defined as *p* < 0.05.

## Results

### The morphological and physiological responses induced by drought stress in wheat seedlings

**Fig. 1:**
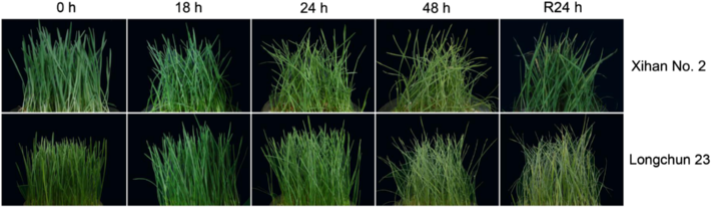
The drought-induced morphological responses in wheat seedlings. The wheat seeds of Xihan No. 2 and Longchun 23 were sown in glass plates containing expanded perlite in an environmentally controlled growth room with 25 ± 2 °C, 70 % relative humidity and 16 h photoperiod (300 μmol m^−2^ · s^−1^ light intensity). One-week-old seedlings were subjected to progressive drought stress up to 48 h. Then, the glass plates were re-watered for the recovery of dehydrated seedlings. The photographs of two wheat cultivars were taken from 0 h, dehydration treatments (18 h, 24 h and 48 h) and rehydration treatment (R24 h), respectively

**Fig. 2:**
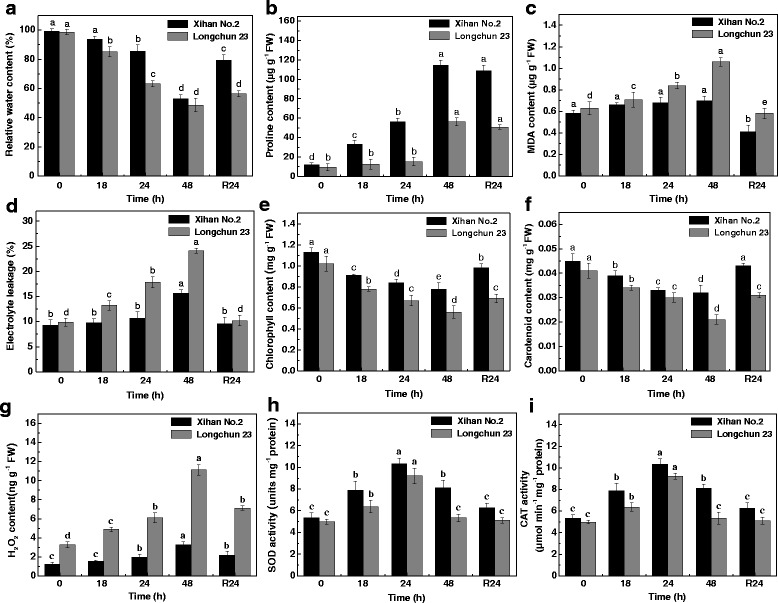
The drought-induced physiological responses in wheat seedlings. The RWC (**a**), free proline content (**b**), MDA content (**c**), electrolyte leakage (**d**), chlorophyll content (**e**), carotenoid content (**f**), H_2_O_2_ content (**g**), SOD activity (**h**) and CAT activity (**i**) were measured from control, dehydration treatments (18 h, 24 h and 48 h) and rehydration treatment (R24 h) of Xihan No. 2 and Longchun 23, respectively. Each value is represented as means ± SE for three independent experiments. Means followed by different small letters are significantly different at *p* < 0.05 according to Duncan’s multiple range test

One-week-old seedlings of two wheat cultivars were subjected to gradual dehydration treatments over 48 h. There were no visible morphological changes in seedlings until 18 h dehydration treatment, but the leaves of both two cultivars began to roll after 24 h, and the damage was further aggravated at 48 h (Fig. 1). After 24 h rehydration, the seedlings of Xihan No. 2 were obviously recovered and no recovery was found in Longchun 23 (Fig. 1). During the whole drought stress period, Xihan No. 2 still showed a higher RWC than Longchun 23 (Fig. 2a). The RWC was significantly declined by 35.8 % in Longchun 23 but only declined by 15.8 % in Xihan No. 2 after 24 h dehydration treatment, and sharply declined in both two cultivars at 48 h. After 24 h rehydration, the RWC of Xihan No. 2 rapidly reached higher value (79.5 %) as compared with Longchun 23 (56.4 %). A rapid accumulation of free proline was observed in Xihan No. 2 after 18 h dehydration treatment, but it was found in Longchun 23 until 48 h (Fig. 2b). After 48 h dehydration treatment, proline content was sharply increased by 8.86-fold in Xihan No. 2 but only increased by 4.99-fold in Longchun 23. MDA and electrolyte leakage as important indexes of membrane injury were measured (Fig. 2c and d). MDA content of Longchun 23 was significantly increased by 68.25 % after 48 h dehydration treatment, whereas no obviously increase was found in Xihan No. 2 (Fig. 2c). It was significantly decreased in both two cultivars after 24 h rehydration. Electrolyte leakage showed a sharp rise in Longchun 23 with the increase of drought stress, whereas there was a significant increase in Xihan No. 2 until 48 h dehydration treatment (Fig. 2d). As compared with a 1.69-fold increase in Xihan No. 2, the increase was occurred in Longchun 23 by 2.44-fold after 48 h dehydration treatment. It was significantly decreased in both two cultivars after 24 h rehydration. The correlation between photosynthetic pigments and drought stress was examined (Fig. 2e and f). Chlorophyll content in both two cultivars was significantly declined during all the stages of drought stress, and the decrease occurred in Longchun 23 by 45.10 % as compared with a decrease only by 30.10 % in Xihan No. 2 after 48 h dehydration treatment (Fig. 2e). Carotenoid content also showed a significant decline in both two cultivars during all the stages of drought stress, and it decreased after 24 h rehydration (Fig. 2f). The oxidative damage induced by drought stress was also examined (Fig. 2g, h and i). The H_2_O_2_ level in Longchun 23 was higher than Xihan No. 2 during all the stages of drought stress (Fig. 2g). H_2_O_2_ content was rapidly increased by 241.41 % in Longchun 23 after 48 h dehydration treatment but only increased by 166.39 % in Xihan No. 2. After 24 h rehydration, H_2_O_2_ content of two cultivars was decreased. The activity of SOD and CAT in both two cultivars was initially increased until 24 h dehydration treatment, and then decreased by 42.02 % and 14.10 % in Longchun 23 at 48 h as compared with a decrease only by 21.22 and 11.26 % in Xihan No. 2, respectively (Fig. 2h and i).

### Identification of drought-responsive proteins by 2-DE and MS in two wheat cultivars

**Fig. 3:**
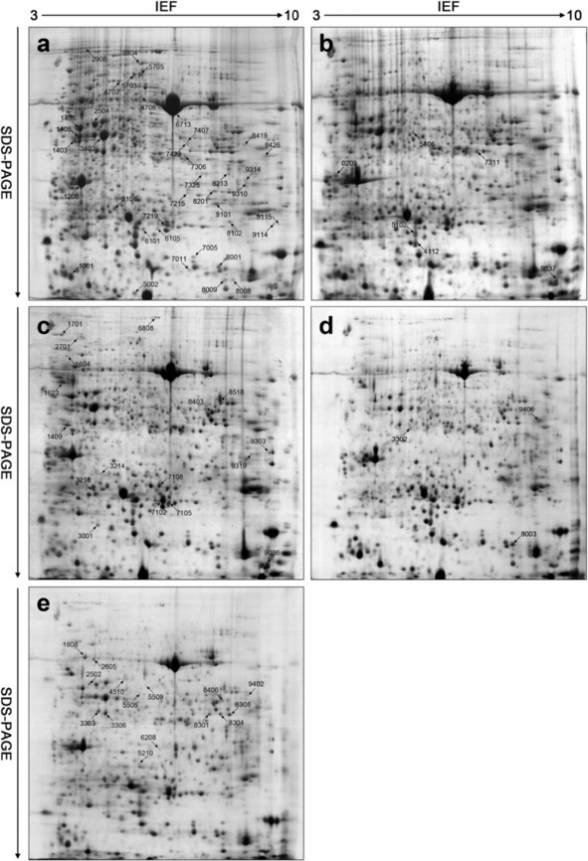
2-DE gel analysis of proteins extracted from leaves of Xihan No. 2 during dehydration and rehydration. Equal amounts (900 μg) of proteins were separated on pH 3–10 IPG strips (17 cm, linear) in the first dimension and by SDS-PAGE on 12 % polyacrylamide gels in the second dimension. The gels were visualized by CBB staining. Three replicate CBB-stained gels for control, dehydration treatments (18 h, 24 h and 48 h) and rehydration treatment (R24 h) (Additional file 1: Figure S3) were computationally combined using PDQuest v8.0.1 software, respectively. Protein spots indicated with numbers were identified by MALDI-TOF/TOF MS. The identified spots were numbered in accordance with Additional file 6: Table S4. **a** 2-DE protein profile for control; (**b**-**e**) 2-DE protein profile for dehydration treatments (18 h, 24 h and 48 h) and rehydration treatment (R24 h), respectively

**Fig. 4:**
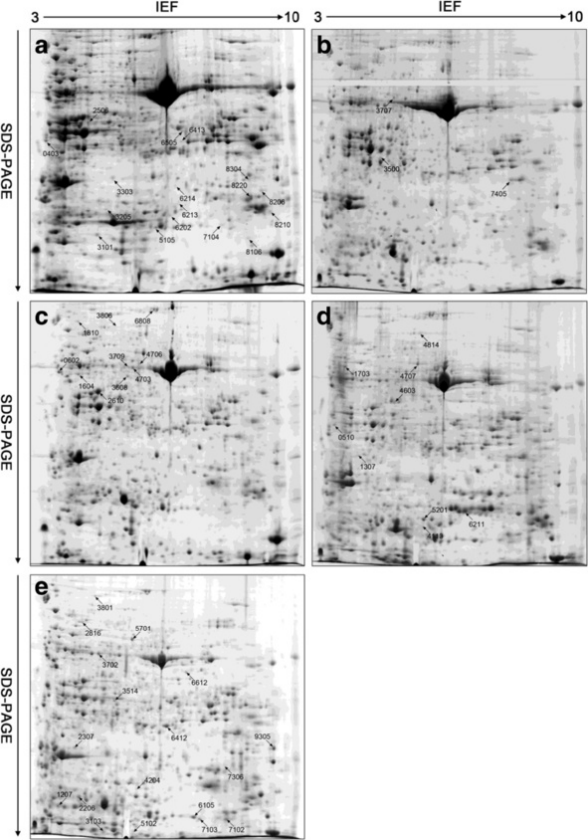
2-DE gel analysis of proteins extracted from leaves of Longchun 23 during dehydration and rehydration. Equal amounts (900 μg) of proteins were separated on pH 3–10 IPG strips (17 cm, linear) in the first dimension and by SDS-PAGE on 12 % polyacrylamide gels in the second dimension. The gels were visualized by CBB staining. Three replicate CBB-stained gels for control, dehydration treatments (18 h, 24 h and 48 h) and rehydration treatment (R24 h) (Additional file 2: Figure S4) were computationally combined using PDQuest v8.0.1 software, respectively. Protein spots indicated with numbers were identified by MALDI-TOF/TOF MS. The identified spots were numbered in accordance with Additional file 7: Table S5. **a** 2-DE protein profile for control; (**b**-**e**). 2-DE protein profile for dehydration treatments (18 h, 24 h and 48 h) and rehydration treatment (R24 h), respectively

**Fig. 5:**
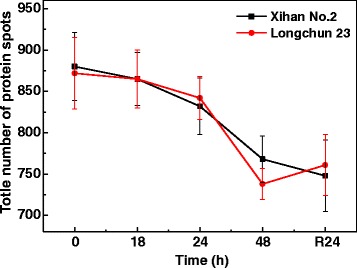
The total number of protein spots detected from the 2-DE gel of Xihan No. 2 and Longchun 23 during dehydration and rehydration

Comparative proteomics analysis was used to investigate the changes of protein profiles in two wheat cultivars under drought stress. Total leaf proteins of control, dehydration treatments (18 h, 24 h and 48 h) and rehydration treatment (R24 h) was extracted and separated by 2-DE, and three replicate gels for control and each treatment were obtaind (Additional file 1: Figure S3, Additional file 2: Figure S4). Figures 3 and 4 showed the representative standard gel maps of Xihan No. 2 and Longchun 23, respectively. The total numbers of protein spots reproducibly detected from control, dehydration treatments (18 h, 24 h and 48 h) and rehydration treatment (R24 h) in Xihan No. 2 were 880 ± 41, 865 ± 32, 832 ± 34, 768 ± 28 and 748 ± 43, respectively (Fig. 5). In Longchun 23, the total numbers of protein spots were 872 ± 43, 865 ± 35, 842 ± 26, 738 ± 19 and 761 ± 37, respectively (Fig. 5). The total number of protein spots on 2-DE gels was gradually declined in both two cultivars during all the stages of drought stress (Fig. 5). Quantitative image analyses showed a total of 172 protein spots from Xihan No. 2 and 215 protein spots from Longchun 23 with their abundance significantly altered (*p* < 0.05) by more than at least 2.5-fold under drought stress and rehydration.

**Fig. 6:**
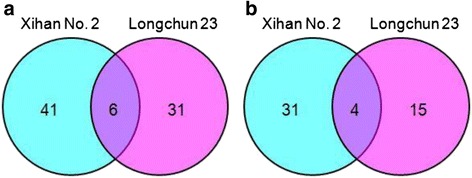
Venn diagrams of the number of up- (**a**) and down-regulated (**b**) proteins in Xihan No. 2 and Longchun 23 under drought stress. Overlapping regions of the circles indicate the number of proteins regulated in either the same manner in both two wheat cultivars, whereas non-overlapping circles indicated proteins regulated in only that cultivar

One hundred and forty-eight differentially abundant proteins were identified by MALDI-TOF/TOF MS in total, including 84 proteins identified in Xihan No. 2 and 64 proteins identified in Longchun 23, respectively. The primary identification information of these differentially abundant proteins of two wheat cultivars were presented in Additional file 3: Table S1, Additional file 4: Table S2 and Additional file 5: Table S3, which were summarised in Additional file 6: Table S4 and Additional file 7: Table S5. To generate a board survey of identified proteins with altered abundance under drought stress, a Venn diagram was conducted to show the dynamics of the number of differentially abundant proteins between Xihan No. 2 and Longchun 23 (Fig. 6). Among these identified proteins, 6 proteins (acid phosphatase, glyceraldehyde-3-phosphate dehydrogenase, peptidyl-prolyl cis-trans isomerase, proteasome subunit alpha, voltage dependent anion channel and S-like RNase) were up-regulated and 4 proteins (ribulose-1,5-bisphosphate carboxylase/oxygenase large subunit, RuBisCO large subunit-binding protein subunit alpha, elongation factor Tu and S-adenosylmethionine synthase) were down-regulated in both two cultivars under drought stress. 41 and 31 proteins were up-regulated only in Xihan No. 2 and Longchun 23, respectively (Fig. 6a). 31 and 15 proteins were down-regulated only in Xihan No. 2 and Longchun 23, respectively (Fig. 6b). Except for the quantitative changes, some proteins also showed qualitative changes in both two cultivars. Five proteins (spots 3508, 3806, 4113, 6214 and 6215) were disappeared after 48 h dehydration treatment, and two proteins (spots 3500 and 6211) absent in control were induced under drought stress in Longchun 23. In Xihan No. 2, two proteins (spots 9037 and 2701) were disappeared after 48 h dehydration treatment. Otherwise, it was noted that the same protein migrated to different gel spots, and their function was common to different spots. In Xihan No. 2, 16 proteins were identified in two to four spots, that is, glyceraldehyde-3-phosphate dehydrogenase (spot 8304 and 8301), putative acid phosphatase (spots 9036 and 9037), putative inactive purple acid phosphatase 27 (spots 5703 and 5705), S-adenosylmethionine synthase (spot 4501, 4506 and 4706), ribulose1,5-bisphosphate carboxylase activase isoform 1 (spots 3402 and 2504), fructose-bisphosphate aldolase (spot 7407, 2309, 3303 and 3306), fructose-bisphosphate aldolase precursor (spots 3302 and 5505), protochlorophyllide reductase (spot 8426 and 9406), glutathione transferase (spots 7102, 6105 and 6101), cyclophilin-like protein (spots 8001 and 8003), germin-like protein 1 (spots 5102, 4112 and 3001), F1-ATPase (spots 9114 and 9115), adenylate kinase A (spot 8201 and 7215), aspartic proteinase nepenthesin-1 precursor (spots 8518, 9310 and 7311), chloroplast stem-loop binding protein of 41 kDa b (spots 8304 and 8308) and S-like RNase (spots 7219 and 7108) (Additional file 3: Table S1, Additional file 6: Table S4). In Longchun 23, 7 proteins were identified in two or three spots, that is ribulose-1,5-bisphosphate carboxylase/oxygenase large subunit (spot 4708 and 4705), glutamate-1-semialdehyde 2,1-aminomutase (spots 3508 and 3511), thaumatin-like protein TLP5 (spots 7104 and 6105), 50S ribosomal protein L10 (spots 3103 and 5102), ATP-dependent Clp protease proteolytic subunit (spots 3205 and 2206), mitochondrial outer membrane porin (spots 8220 and 8250) and rab protein (spots 6212, 6215 and 7206) (Additional file 4: Table S2, Additional file 7: Table S5). The multiple observation of same protein on 2-DE gels could be due to post-translational modifications such as glycosylation, phosphorylation and proteolytic cleavage that can alter the molecular weight and charge of these proteins.

### Functional classification of drought-responsive proteins in two wheat cultivars

**Fig. 7:**
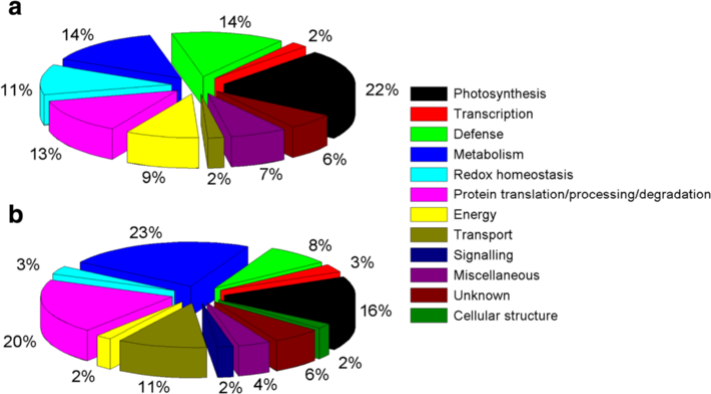
Functional classification of the differentially abundant proteins in Xihan No. 2 (**a**) and Longchun 23 (**b**) during dehydration and rehydration. The protein function classification was conducted according to the putative functions assigned to each of the candidate proteins using the protein functional database and displayed in the pie chart

The identified proteins play a variety of functions during cellular adaptation to drought stress. In Xihan No. 2, 84 differentially abundant proteins were grouped into ten functional classes (Fig. 7a and Additional file 6: Table S4). The largest percentage of identified proteins was involved in photosynthesis (22 %), and the second classes corresponded functions were involved in defence (14 %) and metabolism (14 %). Protein translation/processing/degradation and redox homeostasis accounted 13 % and 11 %, respectively. Proteins were also found to play roles in energy (9 %), miscellaneous (7 %), unknown (6 %), transcription (2 %) and transport (2 %). A wide range of cellular functions were also covered in Longchun 23, which were grouped into twelve functional classes (Fig. 7b and Additional file 7: Table S5). It included metabolism, photosynthesis, protein translation/processing/degradation, redox homeostasis, defence, energy, transcription, cellular structure, signalling, transport, miscellaneous and unknown. The major functional class corresponded proteins involved in metabolism (23 %), protein translation/processing/degradation (20 %), photosynthesis (16 %), transport (11 %) and defence (8 %).

### Dynamics of drought-responsive protein networks in two wheat cultivars

**Fig. 8:**
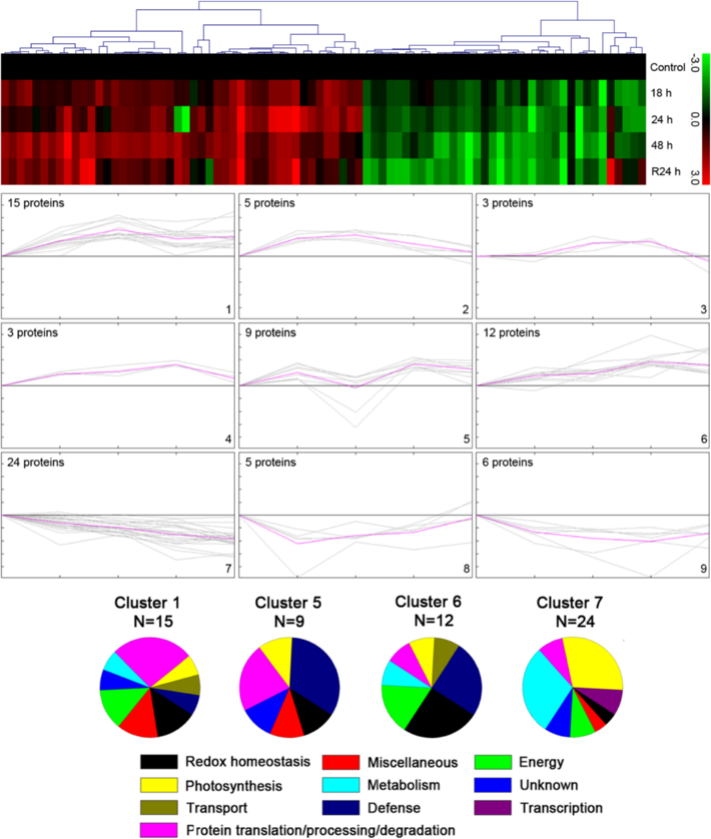
Clustering analysis of the expression profiles of differentially abundant proteins in Xihan No. 2 during dehydration and rehydration. The hierarchical cluster tree is shown at the top, and the expression profiles are shown below. The five rows of hierarchical cluster tree represent control, dehydration treatments (18 h, 24 h and 48 h) and rehydration treatment (R24 h), respectively. Each individual protein is represented by a single column of colour boxes. The up- and down-regulated proteins are indicated in red and green, respectively. The colours intensity is increased with the expression differences increasing, as shown in the bar. The expression profile of each individual protein in the cluster is depicted by gray lines, while the mean expression profile is marked in pink for each cluster. The number of proteins in each cluster is given in the left upper corner, and the cluster number is given in the right lower corner. Only the clusters with n > 6 were taken to investigate the co-expression patterns for functionally similar proteins. The detailed information on proteins within each cluster is presented in Additional file 8: Figure S1

**Fig. 9:**
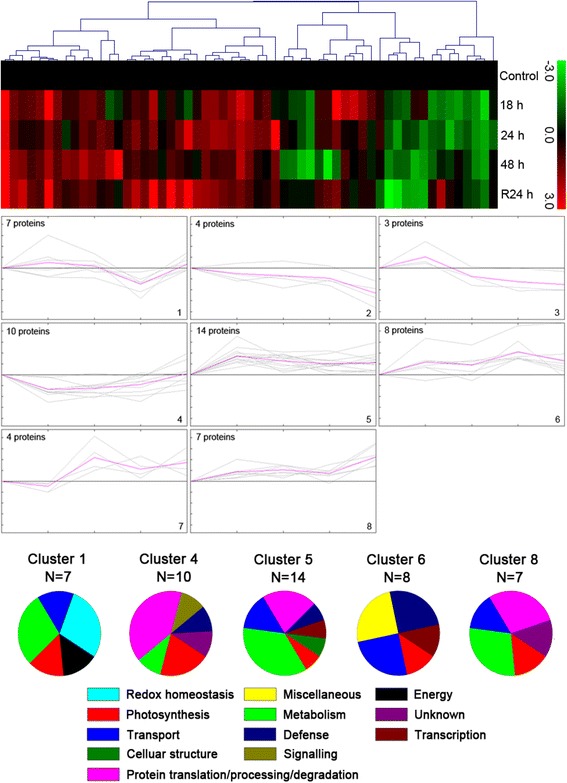
Clustering analysis of the expression profiles of differentially abundant proteins in Longchun 23 during dehydration and rehydration. The hierarchical cluster tree is shown at the top, and the expression profiles are shown below. The five rows of hierarchical cluster tree represent control, dehydration treatments (18 h, 24 h and 48 h) and rehydration treatment (R24 h), respectively. Each individual protein is represented by a single column of colour boxes. The up- and down-regulated proteins are indicated in red and green, respectively. The colours intensity is increased with the expression differences increasing, as shown in the bar. The expression profile of each individual protein in the cluster is depicted by gray lines, while the mean expression profile is marked in pink for each cluster. The number of proteins in each cluster is given in the left upper corner, and the cluster number is given in the right lower corner. Only the clusters with *n* > 6 were taken to investigate the co-expression patterns for functionally similar proteins. The detailed information on proteins within each cluster is presented in Additional file 9: Figure S2

To summarize the proteins with similar expression profiles listed in Additional file 6: Table S4 and Additional file 7: Table S5, the hierarchical clustering was applied to differentially abundant proteins identified in two wheat cultivars. The clustering analysis yielded nine and eight expression clusters in Xihan No. 2 and Longchun 23 (Figs. 8 and 9), respectively. The detailed information on proteins within each cluster is presented in Additional file 8: Figure S1 and Additional file 9: Figure S2. The proteins involved in redox homeostasis, defense, energy and protein translation/processing/degradation, played key roles in drought tolerance of Xihan No. 2 (Fig. 8). These proteins showed an early induction for drought response and maintained almost steady state henceforth in Cluster 1 and 6. However, non-homogeneous expression patterns were also observed in proteins with these functions. Cluster 5 enriched in defense and protein translation/processing/degradation-related proteins were firstly up-regulated and followed by a gradual down-regulation after 18–24 h drought stress, and then induced again until recovery. The co-clustering pattern was also found for unknown proteins in Cluster 1 and 5. Identification of these proteins might provide some valuable insight into kinetics of drought tolerance mechanisms. The most abundant group, Cluster 7 with 24 proteins, were found to be down-regulated during all the stages of drought stress, showing the maximum co-clustering for the proteins involved in photosynthesis and metabolism. Due to heterogeneous composition, the miscellaneous category of proteins were represented in almost all the clusters and showed no clear clustering patterns. In Longchun 23, Cluster 1 was early drought-responsive and showed down-regulation after 24 h drought stress (Fig. 9), which was enriched in proteins associated with metabolism, redox homeostasis, photosynthesis, energy and transport. The proteins involved in protein translation/processing/degradation and photosynthesis as the major functional classes in Cluster 4 were observed to be down-regulated during all the stages of drought stress and recovered after rehydration. Cluster 5 involved in metabolism, protein translation/processing/degradation and transport was early induced and maintains almost steady state henceforth. The other two major groups were Cluster 6 and 8. The proteins in Cluster 6 were gradually up-regulated and involved in transport and defence. The metabolism and protein translation/processing/degradation-related proteins in Cluster 8 were early induced and maintain almost steady state during 18–48 h drought stress, and then up-regulated after rehydration. The co-clustering pattern was also found for unknown proteins in Cluster 8. Proteins involved in photosynthesis and metabolism showed no clear clustering patterns.

## Discussion

Drought is the most important limiting factor for wheat production, and it is becoming an increasingly severe problem in northwestern regions of China. In addition to the complexity of drought itself, the responses of different wheat genotype to drought are complex. The different mechanisms are adopted by wheat genotype with different drought tolerance when they encounter drought stress. The present research about physiology and comparative proteomics in two wheat cultivars with different drought tolerance will help to establish the precise screening techniques to identify traits which are related to drought tolerance in wheat.

### Metabolism-related proteins

Drought can often cause significant metabolism alteration in plants so as to produce some important metabolic intermediates and more energy against drought stress [21, 53, 54]. Several key enzymes involved in glycolysis pathway were up-regulated under drought stress, that is, glyceraldehyde-3-phosphate dehydrogenase (spot 8403) in Xihan No. 2, glyceraldehyde-3-phosphate dehydrogenase A (spot 6413), triosephosphate isomerase (spot 1207) and enolase 2 (spot 3702) in Longchun 23. Glyceraldehyde-3-phosphate dehydrogenase (GAPDH) is responsible for the interconversion of 1,3-diphosphoglycerate and glyceraldehyde-3-phosphate, a central step in glycolysis and gluconeogenesis [55, 56]. The up-regulation of GAPDH in both two wheat cultivars can promote glucose metabolism to meet the increased substance and energy requirement for drought resistance as suggested by Rochat et al. [57]. Triosephosphate isomerase (TPI) catalyzes the reversible interconversion of dihydroxyacetone phosphate and D-glyceraldehyde 3-phosphate, which is essential for efficient energy production in glycolysis [58]. The up-regualtion of TPI (spot 1207) in Longchun 23 is consistent with the observation in rice and maize under drought stress [59, 60]. Enolase 2 (spot 3702) catalyzes the dehydration of 2-phosphoglycerate to phosphoenolpyruvate, which has been reported in response to salt, drought, cold and anaerobic stress [33, 48, 59, 61]. It suggested that the strengthened glycolysis pathway can lead to acetyl-CoA accumulation in Krebs cycle and finally produce a large amount of ATP for drought resistance. Malate dehydrogenase (spots 6412 and 7405) associated with Krebs cycle was also up-regulated during the early period of drought stress in Longchun 23, which might accelerate Krebs cycle for drought adaption [33, 62]. In addition, two crucial enzymes in pentose phosphate pathway (PPP), 6-phosphogluconate dehydrogenate (spot 3608) and transketolase (spot 3806), were up-regulated in Longchun 23. It was most likely that PPP may be another pathway for producing more energy in response to drought [33]. Such a number of regulated glucose metabolism-related enzymes in Longchun 23 reflected an increased energy requirement in drought-sensitive cultivar than tolerant cultivar under drought stress.

Drought can cause changes in free amino acid levels in plant cells [21, 33, 63]. There were several amino acid biosynthesis-related enzymes affected by drought stress in two wheat cultivars. Glutamine synthetase (GS) is responsible for the first step of ammonium assimilation and transformation into glutamine and proline (an important osmolyte) precursors [64, 65]. The up-regulated plastid glutamine synthetase isoform GS2c (spot 1408) in Xihan No. 2 can lead to proline accumulation and enhance osmotic adjustment ability of cells under drought stress as suggested by Díaz et al. [66]. It appeared that proline biosynthesis may be an important amino acid metabolism strategy against drought in drought-tolerant cultivar. S-adenosylmethionine synthase (SAMS) catalyzes a conjugation of methionine and ATP to generate SAM [67, 68]. Previous study has reported that SAMS plays a role in betaine (an organic osmolyte) biosynthesis induced by drought [69] and promotes lignin accumulation for the rearrangement and reinforcement of cell wall [70, 71]. The up-regulated SAMS 2 (spot 4603) in Longchun 23 during the early period of drought stress may contribute to the early protection mechanism against drought through osmolyte accumulation or an accelerated formation of vascular tissue and aerenchyma.

There were also other metabolism-related enzymes providing additional information for wheat in response to drought stress. Previous studies have found that acid phosphatase (ACP) activity was increased under low phosphorus stress [72–74]. The up-regulation of acid phosphatase 1 (spot 8210) in Longchun 23 and putative acid phosphatase (spots 9036 and 9037) in Xihan No. 2 during the early period of drought stress indicated that phosphate metabolism may be a positive drought-response by promoting phosphate absorption, transport and utilization in wheat. Aldehyde dehydrogenases (ALDHs) belong to a family of NAD(P)^+^-dependent enzymes that catalyze the oxidation of various toxic aldehydes to carboxylic acids [75, 76]. The up-regulation of ALDH family 2 member B7 (spot 4703) in Longchun 23 during the early period of drought stress can decrease the toxicity of aldehyde supported from the report of Sunkar et al. [77]. Formate dehydrogenase (FDH) is a mitochondrial and NAD-dependent enzyme that catalyzes the oxidation of formate to carbon dioxide in plants [78, 79]. Previous study has demonstrated that FDH activity was highest in intact sprouting potato tubers under hypoxia stress [80]. The up-regulated FDH (spot 7401) in Xihan No. 2 during the middle period of drought stress reflected its important role in anaerobic metabolism of drought-tolerant cultivar.

### Photosynthesis-related proteins

Oxygen-evolving enhancer proteins (OEE) as an auxiliary component of photosystem II (PS II) manganese cluster can control O_2_ evolution and maintain the stability of PS II [81, 82]. The gradually down-regulated OEE 1–2 (spot 1208) in Xihan No. 2 and the rapidly down-regulated OEE 2 (spot 4113) in Longchun 23 under drought stress suggested that drought-tolerant cultivar have more stability of oxygen-evolving activity of PS II. Rubisco is a key rate-limiting enzyme responsible for photosynthetic carbon assimilation [83, 84]. There were several down-regulated Rubisco proteins found in two wheat cultivars, including Ribulose-1,5-bisphosphate carboxylase/ oxygenase (RuBisCO) large subunit (spot 6713), Ribulose1,5-bisphosphate carboxylase activase isoform 1 (spots 3402 and 2504), RuBisCO large subunit-binding protein subunit alpha (spot 1604) and subunit beta (spot 2908) in Xihan No. 2 and RuBisCO large subunit (spot 4708), RuBisCO large subunit-binding protein subunit alpha (spot 1703), RuBisCO large subunit (spot 4705) and rbcL (spot 6214) in Longchun 23. It might be one of main non-stomatal factors for the decreased photosynthetic rate in two wheat cultivars under drought stress as suggested by Galmés et al. [85]. Fructose-1, 6-bisphosphate aldolase (FBA) reversibly catalyzes the conversion of fructose-1,6-bisphosphate (FBP) to glyceraldehyde 3-phosphate (GAP) and dihydroxy acetone 3-phosphate [86, 87]. Under stress condition, both GAP and FBP may be converted to glucose 6-phosphate for re-entry into the PPP for NADPH synthesis [63]. The down-regulation of FBA (spots 7407, 2309, 3303, 3306, 3302 and 5505) in Xihan No. 2 can enhance NADPH synthesis for energy and maintenance of Calvin cycle. Otherwise, some photosynthetic pigment biosynthesis-regulated enzymes were also identified in two wheat cultivars. Protochlorophyllide reductase (spots 8426 and 9406), catalyzing phototransformation of protochlorophyllide to chlorophyllide in chlorophyll biosynthesis [88, 89], was up regulated in Xihan No. 2. Glutamate-1-semialdehyde 2,1-aminomutase (spots 3508 and 3511) participating in porphyrin and chlorophyll metabolism [90] and magnesium-protoporphyrin O-methyltransferase (spot 7306) involved in light-independent chlorophyll biosynthesis [91] were up-regulated in Longchun 23. It suggested that the enhanced photosynthetic pigment synthesis might be a common mechanism in response to drought stress in both drought-tolerant and sensitive cultivars.

### Redox homeostasis-related proteins

Plants have developed some antioxidative systems including various antioxidants and antioxidase to protect against oxidative damage caused by reactive oxygen species (ROS) under drought stress [20, 41, 92]. Several antioxidases were found to be up-regulated in Xihan No. 2 under drought stress, including glutathione transferase (spots 7102, 6105 and 6101), glutathione peroxidase-like protein GPX54Hv (spot 7005), peroxidase (spots 9314 and 8213), probable L-ascorbate peroxidase 6 (spot 6208) and manganese superoxide dismutase (Mn-SOD, spot 7105). Glutathione transferases (GSTs) as important detoxification enzymes catalyze the conjugation of xenobiotics or their metabolites to glutathione (GSH) [93, 94]. Glutathione peroxidases (GPXs) catalyze the reduction of H_2_O_2_, organic hydroperoxides and lipid peroxides using GSH and/or other reducing equivalents [95]. The up-regulation of GSTs (spots 7102, 6105 and 6101) and GPX-like protein GPX54Hv (spot 7005) in Xihan No. 2 may protect cell membrane from oxidative damage and maintain cellular redox homeostasis [96, 97]. The up-regulated probable L-ascorbate peroxidase 6 (spot 6208) in Xihan No. 2 can detoxify H_2_O_2_ to H_2_O and promote the fine-tuning of ascorbate-glutathione cycle [98, 99]. Mn-SOD (spot 7105) is the principal scavenger for superoxide in mitochondria, thus its up-regulation in Xihan No. 2 may provide the dismutation role of superoxide radical to hydrogen peroxide and oxygen in mitochondrial [100, 101]. It appeared that the multi-components antioxidant systems may take part in ROS scavenging and maintain a higher drought tolerance in Xihan No. 2. However, only one up-regulated ascorbate peroxidase (spot 5201) and one down-regulated gamma-glutamylcysteine synthetase (γ-GCS, spot 2602) catalyzing production of the cellular antioxidant GSH [102, 103] were identified in Longchun 23. The down-regulation of γ-GCS might inhibit GSH synthesis and lead to a higher oxidative state in Longchun 23 under drought stress.

### Defense-related proteins

Twelve defense-related proteins were identified in Xihan No. 2, including glucan endo-1,3-beta-glucosidase (spot 9319), cyclophilin-like protein (spots 8001 and 8003), NAD(P)-binding Rossmann-fold-containing protein (spot 5210), CBS domain containing protein (spot 8009), alpha-1,4-glucan-protein synthase (spot 5406), germin-like protein 1 (spots 5102, 4112 and 3001), xylanase inhibitor TAXI-IV (spot 9402), stress responsive protein (spot 7306) and USP family protein (spot 5002). Except for alpha-1,4-glucan-protein synthase and germin-like protein 1, all the other proteins were up-regulated at least one time stage under drought stress. Glucan endo-1,3-beta-glucosidase (spot 9319) can degrade the fungal cell wall polysaccharides [104, 105], and its up-regulation in Xihan No. 2 may protect against fungal pathogen infection under drought stress. Cyclophilin-like protein (spots 8001 and 8003) belongs to a large family of enzyme with peptidyl prolyl isomerase activity, which might participate in stress response and pathogen immunity in Xihan No. 2 as suggested by Chen et al. [106] and Gan et al. [107]. Xylanase inhibitor TAXI-IV (spot 9402) can suppress microbial xylanases and participate in defense against fungal and bacteria pathogens [108, 109]. CBS domain containing protein (spot 8009) is connected by 2 or 4 cystathionine β-synthase (CBS) domains [110]. Over-expression of this protein can improve salinity, oxidative and heavy metal tolerance in transgenic tobacco [111], speculating its important role in stress response. NAD(P)-binding Rossmann-fold-containing protein (spot 5210) can produce coenzyme NAD(P)^+^ that is an important proton transfer and energy receptor in respiration, and its up-regulation in Xihan No. 2 may accelerate anaerobic respiration and reduce toxic substances accumulation under drought stress. In addition, the up-regulation of stress responsive protein (spot 7306) and USP family protein (spot 5002) also showed the potential to improve resistance against drought in Xihan No. 2. However, only a few defense-related proteins were identified in Longchun 23, such as putative plastid-lipid-associated protein 3 (spot 0403) involved in drought-related jasmonate biosynthesis [112], thaumatin-like protein TLP5 (spots 7104 and 6105) and class II chitinase-like protein (spot 3301) associated with pathogen resistance [113, 114]. All the results indicated that more defense mechanisms were induced in the drought-tolerant cultivar than in the sensitive cultivar under drought stress, which can contribute to stronger drought resistance.

### Energy-related proteins

F-ATPase is a key enzyme of energy metabolism that uses the transmembrane electrochemical proton gradient generated by oxidative phosphorylation or photosynthesis to drive ATP synthesis [115, 116]. The up-regulation of F1-ATPase (spots 9114 and 9115) and ATP synthase precursor (spot 3108) in Xihan No. 2 might enhance ATP synthesis and provide more energy for drought resistance. The similar behaviours of these proteins were also described in wheat, rice, *Boea hydrometrica* and *Arabidopsis thaliana* under various abiotic stresses [92, 117–119]. The up-regulated adenylate kinase A (spots 8201 and 7215) in Xihan No. 2 could contribute to regulate multiple cellular energy-dependent and nucleotide signaling processes under drought stress through catalyzing phosphotransfer as suggested by Dzeja and Terzic [120]. However, only one energy-related protein, F0-F1 ATPase alpha subunit (spot 3707), were identified in Longchun 23, which was up-regulated during the early period of drought stress and then down-regulated. It suggested that comparing to a transient enhancement of ATP synthesis during the early period of drought stress in sensitive cultivar, drought-tolerant cultivar can enhance energy metabolism for drought resistance through a continuous ATP synthesis.

### Protein translation, processing and degradation-related proteins

Many identified proteins in two wheat cultivars were attributed to protein metabolism, which were divided into three functional groups. The first group was functioned in protein biosynthesis. Translation elongation factor is a core translational protein that catalyzes the initiation and elongation of newly growing peptide chains [121, 122]. Several GTP-driven elongation factors, chloroplast translational elongation factor Tu (spot 2502) in Xihan No. 2 and elongation factor G (spot 1810) and elongation factor Tu (spot 2610) in Longchun 23, were down-regulated, reflecting that the GTP-dependent ribosomal translocation elongation of protein biosynthesis might be inhibited by drought stress [123, 124]. The similar behaviours of these proteins were also described in soybean genotypes with different salt tolerance [125]. Ribosomal protein is one kind of highly conserved proteins that make up ribosomal subunits involved in the cellular process of translation [126]. The up-regulation of 30S ribosomal protein S5 (spot 1409) in Xihan No. 2 and 50S ribosomal protein L10 (spots 3103 and 5102) in Longchun 23 can promote mRNA/ribosome interactions early in translation [127]. The second group participated in protein degradation. Proteolysis is necessary for the removal of abnormal, modified and mistargeted proteins and for altering the balance of proteins [21]. Proteasome subunit alpha type-2 (spot 3218), 20S proteasome beta 7 subunit (spot 8102), aspartic proteinase nepenthesin-1 precursor (spots 8518, 9310 and 7311), triticain alpha (spot 0209) in Xihan No. 2, and proteasome subunit alpha type-7-A (spot 8304), proteasome subunit alpha type-1 (spot 1307) and ATP-dependent Clp protease proteolytic subunit (spots 3205 and 2206) in Longchun 23, were up-regulated under drought stress. It was postulated that proteases and proteasomes play key roles in maintaining strict protein quality control and degrading specific sets of proteins in response to drought stress in both drought-tolerant and sensitive cultivars [21, 128, 129]. The third group was engaged in protein refolding and assembly. Heat shock proteins (HSPs) act as molecular chaperones that protect plants against various stresses by re-establishing normal protein conformation and cellular homeostasis [130–132]. Two HSP70s, 70 kDa heat shock protein (spot 1701) and chloroplast envelope membrane 70 kDa heat shock-related protein (spot 2701), were down-regulated in Xihan No. 2. It was proposed that the transport of newly synthesized peptides was decreased in Xihan No. 2, partially due to the decreased protein synthesis under stress conditions as suggested by Jiang et al. [133] and Cheng et al. [134]. Chaperone protein dnaJ 10 (spot 6505) as an Hsp40 chaperone was up-regulated in Longchun 23 during the middle period of drought stress, which can help transfer substrate proteins to Hsp70s and regulate their ATPase activity [135]. The down-regulated chaperone protein ClpC1 (spot 3801) in Longchun 23 can reduce the degradation ability of denatured chloroplast proteins. Peptidyl-prolyl cis-trans isomerases, a superfamily of ubiquitous folding catalysts, catalyzes the interconversion of peptidyl-prolyl imide bonds in peptide and protein substrates [136, 137]. The up-regulated peptidyl-prolyl cis-trans isomerase FKBP20-2 (spot 1116) in Xihan No. 2 and down-regulated peptidyl-prolyl cis-trans isomerase (spot 0510) in Longchun 23 reflected its critical role in accelerating protein folding of drought-tolerant cultivar. Overall, the expression patterns of these proteins in three groups indicated that protein biosynthesis might be inhibited by drought stress in both drought-tolerant and sensitive cultivars, whereas the higher degradation ability of denatured proteins as well as the enhanced protein folding may be appeared in tolerant cultivar for drought resistance.

### Transport, cellular structure and signaling-related proteins

Some transport-related proteins were identified to be up-regulated in Xihan No. 2 and Longchun 23, indicating that there exists active transport of ions and metabolites for drought adaptation in both drought-tolerant and sensitive wheat cultivars. Voltage dependent anion channel (VDAC, or mitochondrial outer membrane porin) regulates metabolic and energetic flux across the outer mitochondrial membrane [138, 139]. The up-regulation of VDAC (spot 9303 and 9305) and mitochondrial outer membrane porin (spot 8220 and 8250) could enhance the exchange of ions and molecules between mitochondria and cytosol for maintaining intracellular homeostasis under drought stress. YLP (spot 7325) belonged to the component of vacuolar H^+^-ATPase subunit E in Xihan No. 2 and vacuolar proton-ATPase subunit A (spot 2816) in Longchun 23 were also up-regulated, which may maintain an electrochemical proton gradient across the tonoplast to drive transmembrane transport of ions and metabolites for drought adaptation as suggested by Wang et al. [140]. The up-regulated rab protein (spot 6212, 6215 and 7206) in Longchun 23 may function as regulators in membrane-trafficking pathways [141]. In addition, there were two proteins associated with cellular structure and signaling functions identified in Longchun 23. Actin can form microfilaments that are essential elements of cytoskeleton [142, 143]. The up-regulated actin (spot 2506) in Longchun 23 might be required to adjust cellular behavior in response to drought stress. Calreticulin (CRT) as an abundant Ca^2+^-binding protein maintains the intracellular Ca^2+^ homeostasis and Ca^2+^ signaling pathway [144], which plays a positive role in stress response of plants such as cold, drought and disease [145–147]. The down-regulated CRT (spot 0602) in Longchun 23 appeared that Ca^2+^ signaling transduction was weakened and thus cannot start some key defense reaction in drought-sensitive cultivar.

### Miscellaneous and unknown proteins

In addition to the major protein classes, other important proteins were also identified. UDP-glucuronate decarboxylase 1 (spot 8406) involved in cell wall biosynthesis of plants [148] was up-regulated and then gradually down-regulated in Xihan No. 2, reflecting that the cell wall components of drought-tolerant cultivar were impacted by drought. The up-regulated ankyrin-repeat protein HBP1 (spot 1623) in Xihan No. 2 might be a positive response to drought signaling by mediating protein-protein interactions [149]. The rapidly down-regulated victorin binding protein (spot 6808) in Xihan No. 2 seems to reduce the intracellular toxin [150]. S-like RNases have been reported to be induced by inorganic phosphate-starvation or in response to pathogen infection and mechanical wounding [151, 152]. The up-regulated S-like RNases (spot 7219, 7108 and 6213) in both two wheat cultivars might act as a positive regulator in drought response as suggested by Zheng et al. [153]. Two key enzymes in ubiquitination-proteasomal pathway, ubiquitin-conjugating enzyme 26 (spot 1001) in Xihan No. 2 and NEDD8-conjugating enzyme Ubc12 (spot 8106) in Longchun 23, were up-regulated, implicating that the ubiquitin-dependent protein degradation plays an important role in drought tolerance of wheat as suggested by Zhou et al. [154]. The up-regulated flavoprotein wrbA-like isoform 1 (spot 5105) in Longchun 23 could prevent interaction of the semiquinone with O_2_ and production of superoxide under drought stress [155]. Otherwise, there were some unknown proteins identified, which are subject to further studies for clarifying their contributions in response to drought stress in wheat.

## Conclusions

**Fig. 10:**
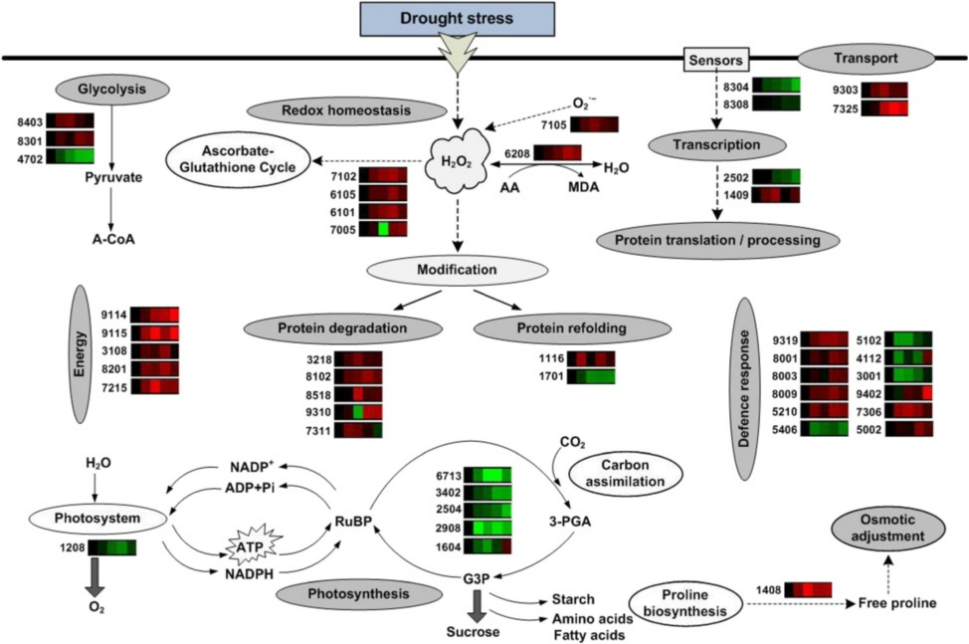
The representative models for summarizing the functional and regulatory networks activated by drought stress in Xihan No. 2. The identified proteins are displayed on the corresponding metabolic pathways. The colour boxes are representative of expression profiles of individual protein during dehydration and rehydration. The up- and down-regulation of proteins are indicated in red and green, respectively. The colours intensity is increased with the expression differences increasing. The number given on the left side of each colour boxes indicates the protein identification number in accordance with Additional file 6: Table S4

**Fig. 11:**
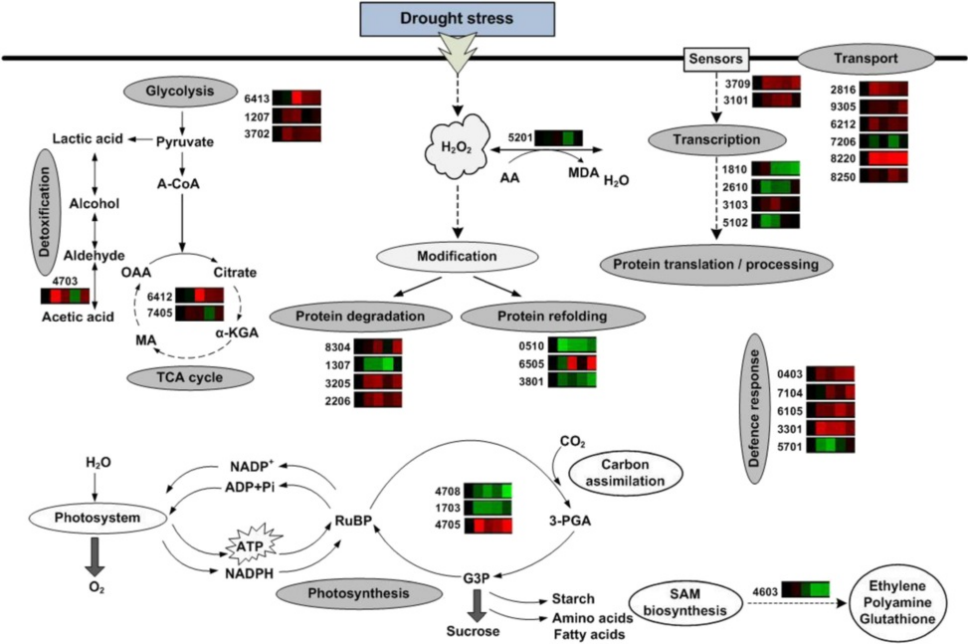
The representative models for summarizing the functional and regulatory networks activated by drought stress in Longchun 23. The identified proteins are displayed on the corresponding metabolic pathways. The colour boxes are representative of expression profiles of individual protein during dehydration and rehydration. The up- and down-regulation of proteins are indicated in red and green, respectively. The colours intensity is increased with the expression differences increasing. The number given on the left side of each colour boxes indicates the protein identification number in accordance with Additional file 7: Table S5

Our integrated analysis of physiology and proteome data provides useful information about the drought-response mechanism of two wheat cultivars with different drought tolerance (Xihan No. 2, a drought-tolerant cultivar and Longchun 23, a drought-sensitive cultivar). Quantitative image analysis of 2-DE gels showed significant variations of 172 protein spots from Xihan No. 2 and 215 protein spots from Longchun 23. Out of these spots, a total of 84 and 64 differentially abundant proteins were identified by MALDI-TOF/TOF MS in Xihan No. 2 and Longchun 23, respectively. Most of these identified proteins were involved in metabolism, photosynthesis, defence and protein translation/processing/degradation in two wheat cultivars. In addition, the proteins involved in redox homeostasis, energy, transcription, cellular structure, signalling and transport were also identified. Hierarchical clustering results revealed that these proteins were involved in a dynamic network in response to drought stress. The representative models for summarizing the functional and regulatory networks activated by drought stress in Xihan No. 2 and Longchun 23 were illustrated in Figs. 10 and 11, respectively. Wheat leaf cells can perceive drought signalling through putative sensors and transmit them to regulate transcription, protein synthesis and processing, thereby affecting the levels of functional proteins involved in metabolism, photosynthesis, redox homeostasis, defence, energy and so on. These cellular processes work more cooperatively to re-establish homeostasis in drought-tolerant cultivar than sensitive cultivar. More glucose metabolism-related enzymes regulated in sensitive cultivar suggested more energy requirement in sensitive cultivar under drought stress. The up-regulation of proline biosynthesis-related enzyme can enhance the osmotic adjustment ability of cells in drought-tolerant cultivar. The down-regulation of Rubisco is one of main non-stomatal factors for the decreased photosynthetic rate in both two wheat cultivars, whereas the relative stability of PS II might be an effective method of drought-tolerant cultivar in response to drought stress. The up-regulation of ATP synthesis-related enzymes can enhance energy metabolism in drought-tolerant cultivar for protective and repair reactions. The higher degradation ability of denatured proteins as well as the enhanced protein folding may be associated with stronger drought resistance in tolerant cultivar. More defense mechanisms induced in the tolerant cultivar than in the sensitive cultivar can also contribute to stronger drought resistance. Future work should integrate transcriptomics, proteomics, and metabolomics approaches to gain a comprehensive knowledge of the sophisticated molecular networks of response and acclimation to drought stress in wheat.

## Additional files

Additional file 1: Figure S3. The primary 2-DE gel maps at least three biological replicates for control, dehydration treatments (18 h, 24 h and 48 h) and rehydration treatment (R24 h) in Xihan No. 2. (TIF 1852 kb) Additional file 2: Figure S4. The primary 2-DE gel maps at least three biological replicates for control, dehydration treatments (18 h, 24 h and 48 h) and rehydration treatment (R24 h) in Longchun 23. (TIF 1707 kb) Additional file 3: Table S1. The primary identification information of differentially abundant proteins associated with drought stress response in Xihan No. 2 by MALDI-TOF/TOF MS. (XLSX 77 kb) Additional file 4: Table S2. The primary identification information of differentially abundant proteins associated with drought stress response in Longchun 23 by MALDI-TOF/TOF MS. (XLSX 74 kb) Additional file 5: Table S3. Information of single-peptide-based protein identifications in Xihan No. 2 and Longchun 23. (XLSX 566 kb) Additional file 6: Table S4. Identification of differentially abundant proteins associated with drought stress response in Xihan No. 2. Protein identifications were performed by searching for the *Viridiplantae* index of the NCBInr database using peptide mass fingerprinting (PMF) and MS/MS data from a MALDI-TOF/TOF mass spectrometry analysis. The spot number as given on the 2-DE gel image (shown in Fig. 3), the identified protein name, the source organism, the gene identification number as in GenBank, the number of matched peptides, the statistical score from the database, the sequence coverage (%), the theoretical Mass (kDa)/ pI values retrieved from protein database and the means for relative protein abundance ± standard error (SE) were listed. Spots with a significant differential expression are described as the spot volumes that were significantly different (*p* < 0.05, at least 2.5-fold) in relative abundance. (DOC 485 kb) Additional file 7: Table S5. Identification of differentially abundant proteins associated with drought stress response in Longchun 23. Protein identifications were performed by searching for the *Viridiplantae* index of the NCBInr database using peptide mass fingerprinting (PMF) and MS/MS data from a MALDI-TOF/TOF mass spectrometry analysis. The spot number as given on the 2-DE gel image (shown in Fig. 4), the identified protein name, the source organism, the gene identification number as in GenBank, the number of matched peptides, the statistical score from the database, the sequence coverage (%), the theoretical Mass (kDa)/pI values retrieved from protein database and the means for relative protein abundance ± standard error (SE) were listed. Spots with a significant differential expression are described as the spot volumes that were significantly different (*p* < 0.05, at least 2.5-fold) in relative abundance. (DOC 372 kb) Additional file 8: Figure S1. The detailed information on differentially abundant proteins within each cluster in the clustering analysis of Xihan No. 2. The five columns of hierarchical cluster tree represent control, dehydration treatments (18 h, 24 h and 48 h) and rehydration treatment (R24 h), respectively. Each rows represent individual proteins. The up- and down-regulation of proteins are indicated in red and green, respectively. The intensity of colours is increased when the expression differences increased, as shown in the bar at the top. The differentially abundant proteins were grouped into 9 clusters in Xihan No. 2. The detailed information on these proteins within each cluster is presented, including the protein identification number, protein name and source organism. (TIF 13294 kb) Additional file 9: Figure S2. The detailed information on differentially abundant proteins within each cluster in the clustering analysis of Longchun 23. The five columns of hierarchical cluster tree represent control, dehydration treatments (18 h, 24 h and 48 h) and rehydration treatment (R24 h), respectively. Each rows represent individual proteins. The up- and down-regulation of proteins are indicated in red and green, respectively. The intensity of colours is increased when the expression differences increased, as shown in the bar at the top. The differentially abundant proteins were grouped into 8 clusters in Longchun 23. The detailed information on these proteins within each cluster is presented, including the protein identification number, protein name and source organism. (TIF 10684 kb)

## Abbreviations

2-DE: Two-dimensional gel electrophoresis
ABA: Abscisic acid
ABF: ABA-binding factor
ACP: Acid phosphatase
ALDHs: Aldehyde dehydrogenases
APX: Ascorbate peroxidise
AREB: ABA-responsive element binding protein
CAT: Catalase
CBB: Coomassie brilliant blue
CBF: C-repeat-binding factor
CBS: Cystathionine β-synthase
CRT: Calreticulin
DHN: Dehydrin
DMAB: 3-dimethyaminobenzoic acid
DREB: Dehydration-responsive element binding protein
DW: Dry weight
FBA: Fructose-1,6-bisphosphate aldolase
FBP: Fructose-1,6-bisphosphate
FDH: Formate dehydrogenase
FW: Fresh weight
GAP: Glyceraldehyde-3-phosphate
GAPDH: Glyceraldehyde-3-phosphate dehydrogenase
GPX: Glutathione peroxidise
GR: Glutathione reductase
GS: Glutamine synthetase
GSH: Glutathione
GST: Glutathione S-transferase
HSPs: Heat shock proteins
IP_3_: 1,4,5-triphosphate
MAPK: Mitogen-activated protein kinase
MBTH: 3-methyl, 2-benzo thiazolinone hydrazone
MDA: Malonaldehyde
MYB: Myeloblastosis oncogene
MYC: Myelocytomatosis oncogene
NBT: Nitroblue tetrazolium
OEE: Oxygen-evolving enhancer
POX: Peroxidase
PPP: Pentose phosphate pathway
PS II: Photosystem II
PVP: Polyvinypyrrolidone
ROS: Reactive oxygen species
RuBisCO: Ribulose-1,5-bisphosphate carboxylase/oxygenase
RWC: Relative water content; weight
SAMS: S-adenosylmethionine synthase
SE: Standard error
SOD: Superoxide dismutase
TBA: Thiobarbituric acid
TPI: Triosephosphate isomerise
TW: Turgid weight
UXS: UDP-glucuronate decarboxylase
VDAC: Voltage dependent anion channel
γ-GCS: Gamma-glutamylcysteine synthetase

## References

[1] Wang W, Vinocur B, Altman A (2003). Plant responses to drought, salinity and extreme temperatures: towards genetic engineering for stress tolerance. Planta.

[2] Tardif G, Kane NA, Adam H, Labrie L, Major G, Gulick P, Sarhan F, Laliberté JF (2007). Interaction network of proteins associated with abiotic stress response and development in wheat. Plant Mol Biol.

[3] The International Wheat Genome Sequencing Consortium (IWGSC) (2014). A chromosome-based draft sequence of the hexaploid bread wheat (*Triticum aestivum*) genome. Science.

[4] Wilkinson S, Davies WJ (2010). Drought, ozone, ABA and ethylene: new insights from cell to plant to community. Plant Cell Environ.

[5] Lee SC, Luan S (2012). ABA signal transduction at the crossroad of biotic and abiotic stress responses. Plant Cell Environ.

[6] Hossain Z, Nouri MZ, Komatsu S (2012). Plant cell organelle proteomics in response to abiotic stress. J Proteome Res.

[7] Chaves MM, Maroco JP, Pereira JS (2003). Understanding plant responses to drought from genes to the whole plant. Funct Plant Biol.

[8] Mahajan S, Tuteja N (2005). Cold, salinity and drought stresses: an overview. Arch Biochem Biophys.

[9] Bhushan D, Pandey A, Choudhary MK, Datta A, Chakraborty S, Chakraborty N (2007). Comparative proteomics analysis of differentially expressed proteins in chickpea extracellular matrix during dehydration stress. Mol Cell Proteomics.

[10] Reddy AR, Chaitanya KV, Vivekanandan M (2004). Drought-induced responses of photosynthesis and antioxidant metabolism in higher plants. J Plant Physiol.

[11] Zang X, Komatsu S (2007). A proteomic approach for identifying osmotic-stress-related proteins in rice. Phytochemistry.

[12] Cui SX, Hu J, Yang B, Shi L, Huang F, Tsai SN (2009). Proteomic characterization of *Phragmites communis* in ecotypes of swamp and desert dune. Proteomics.

[13] Ramanjulu S, Bartels D (2002). Drought- and desiccation-induced modulation of gene expression in plants. Plant Cell Environ.

[14] Bartels D, Sunkar R (2005). Drought and salt tolerance in plants. Crit Rev Plant Sci.

[15] Sakuma Y, Maruyama K, Osakabe Y, Qin F, Seki M, Shinozaki K (2006). Functional analysis of an Arabidopsis transcription factor, DREB2A, involved in drought-responsive gene expression. Plant Cell.

[16] Nakashima K, Ito Y, Yamaguchi-Shinozaki K (2009). Transcriptional regulatory networks in response to abiotic stresses in *Arabidopsis* and grasses. Plant Physiol.

[17] Morran S, Eini O, Pyvovarenko T, Parent B, Singh R, Ismagul A (2011). Improvement of stress tolerance of wheat and barley by modulation of expression of DREB/CBF factors. Plant Biotechnol J.

[18] Xue GP, Way HM, Richardson T, Drenth J, Joyce PA, McIntyre CL (2011). Overexpression of *TaNAC69* leads to enhanced transcript levels of stress up-regulated genes and dehydration tolerance in bread wheat. Mol Plant.

[19] Rahaie M, Xue GP, Naghavi MR, Alizadeh H, Schenk PM (2010). A MYB gene from wheat (*Triticum aestivum* L.) is up-regulated during salt and drought stresses and differentially regulated between salt-tolerant and sensitive genotypes. Plant Cell Rep.

[20] Pandey A, Chakraborty S, Datta A, Chakraborty N (2008). Proteomics approach to identify dehydration responsive nuclear proteins from chickpea (*Cicer arietinum* L.). Mol Cell Proteomics.

[21] Choudhary MK, Basu D, Datta A, Chakraborty N, Chakraborty S (2009). Dehydration-responsive nuclear proteome of rice (*Oryza sativa* L.) illustrates protein network, novel regulators of cellular adaptation, and evolutionary perspective. Mol Cell Proteomics.

[22] Timperio AM, Egidi G, Zolla L (2008). Proteomics applied on plant abiotic stresses: Role of heat shock proteins (HSP). J Proteomics.

[23] Rorat T (2006). Plant dehydrins - tissue location, structure and function. Cell Mol Biol Lett.

[24] Lopez CG, Banowetz GM, Peterson CJ, Kronstad WE (2003). Dehydrin expression and drought tolerance in seven wheat cultivars. Crop Sci.

[25] Rampino P, Pataleo S, Gerardi C, Mita G, Perrotta C (2006). Drought stress response in wheat: physiological and molecular analysis of resistant and sensitive genotypes. Plant Cell Environ.

[26] Muñoz-Mayor A, Pineda B, Garcia-Abellán JO, Antón T, Garcia-Sogo B, Sanchez-Bel P (2012). Overexpression of dehydrin tas14 gene improves the osmotic stress imposed by drought and salinity in tomato. J Plant Physiol.

[27] Imamura T, Higuchi A, Takahashi H (2013). Dehydrins are highly expressed in overwintering buds and enhance drought and freezing tolerance in *Gentiana triflora*. Plant Sci.

[28] Vaseva II, Anders I, Feller U (2014). Identification and expression of different dehydrin subclasses involved in the drought response of *Trifolium repens*. J Plant Physiol.

[29] Mohammadi M, Kav NN, Deyholos MK (2008). Transcript expression profile of water-limited roots of hexaploid wheat (*Triticum aestivum* ‘Opata’). Genome.

[30] Aprile A, Mastrangelo AM, De Leonardis AM, Galiba G, Roncaglia E, Ferrari F (2009). Transcriptional profiling in response to terminal drought stress reveals differential responses along the wheat genome. BMC Genomics.

[31] Ergen NZ, Budak H (2009). Sequencing over 13000 expressed sequence tags from six subtractive cDNA libraries of wild and modern wheats following slow drought stress. Plant Cell Environ.

[32] Wang J, Ding B, Guo Y, Li M, Chen S, Huang G (2014). Overexpression of a wheat phospholipase D gene, *TaPLDα*, enhances tolerance to drought and osmotic stress in *Arabidopsis thaliana*. Planta.

[33] Manaa A, Ben Ahmed H, Valot B, Bouchet JP, Aschi-Smiti S, Causse M (2011). Salt and genotype impact on plant physiology and root proteome variations in tomato. J Exp Bot.

[34] Mohammadi M, Kav NN, Deyholos MK (2007). Transcriptional profiling of hexaploid wheat (*Triticum aestivum* L.) roots identifies novel, dehydration-responsive genes. Plant Cell Environ.

[35] Ergen NZ, Thimmapuram J, Bohnert HJ, Budak H (2009). Transcriptome pathways unique to dehydration tolerant relatives of modern wheat. Funct Integr Genomics.

[36] Sečenji M, Lendvai Á, Miskolczi P, Kocsy G, Gallé Á, Szucs A (2010). Differences in root functions during long-term drought adaptation: comparison of active gene sets of two wheat genotypes. Plant Biol (Stuttg).

[37] Aprile A, Havlickova L, Panna R, Marè C, Borrelli GM, Marone D (2013). Different stress responsive strategies to drought and heat in two durum wheat cultivars with contrasting water use efficiency. BMC Genomics.

[38] Fleury D, Jefferies S, Kuchel H, Langridge P (2010). Genetic and genomic tools to improve drought tolerance in wheat. J Exp Bot.

[39] Pradet-Balade B, Boulme F, Beug H, Muliner EW, Garcia-Sanz JA (2001). Translation control: bridging the gap between genomics and proteomics?. Trends Biochem Sci.

[40] Agrawal L, Chakraborty S, Jaiswal DK, Gupta S, Datta A, Chakraborty N (2008). Comparative proteomics of tuber induction, development and maturation reveal the complexity of tuberization process in potato (*Solanum tuberosum* L.). J Proteome Res.

[41] Ford KL, Cassin A, Bacic A (2011). Quantitative proteomic analysis of wheat cultivars with differing drought stress tolerance. Front Plant Sci.

[42] Bazargani MM, Sarhadi E, Bushehri AA, Matros A, Mock HP, Naghavi MR (2011). A proteomics view on the role of drought-induced senescence and oxidative stress defense in enhanced stem reserves remobilization in wheat. J Proteomics.

[43] Ge P, Ma C, Wang S, Gao L, Li X, Guo G (2012). Comparative proteomic analysis of grain development in two spring wheat varieties under drought stress. Anal Bioanal Chem.

[44] Hao P, Zhu J, Gu A, Lv D, Ge P, Chen G (2014). An integrative proteome analysis of different seedling organs in tolerant and sensitive wheat cultivars under drought stress and recovery. Proteomics.

[45] Faghani E, Gharechahi J, Komatsu S, Mirzaei M, Khavarinejad RA, Najafi F (2015). Comparative physiology and proteomic analysis of two wheat genotypes contrasting in drought tolerance. J Proteomics.

[46] Bates LS, Waldren RP, Teare ID (1973). Rapid determination of free proline for water stress studies. Plant Soil.

[47] Hodges DM, DeLong JM, Forney CF, Prange RK (1999). Improving the thiobarbituric acid-reactive-substances assay for estimating lipid peroxidation in plant tissues containing anthocyanin and other interfering compounds. Planta.

[48] Yan SP, Zhang QY, Tang ZC, Su WA, Sun WN (2006). Comparative proteomic analysis provides new insights into chilling stress responses in rice. Mol Cell Proteomics.

[49] Veljovic-Jovanovic S, Noctor G, Foyer CH (2002). Are leaf hydrogen peroxide concentrations commonly overestimated? The potential influence of artefactual interference by tissue phenolics and ascorbate. Plant Physiol Biochem.

[50] Beyer WF, Fridovich I (1987). Assaying for superoxide dismutase activity: some large consequences of minor changes in conditions. Anal Biochem.

[51] Aebi H (1984). Catalase *in vitro*. Methods Enzymol.

[52] Donnelly BE, Madden RD, Ayoubi P, Porter DR, Dillwith JW (2005). The wheat (*Triticum aestivum* L.) leaf proteome. Proteomics.

[53] Chaves MM, Flexas J, Pinheiro C (2009). Photosynthesis under drought and salt stress: regulation mechanisms from whole plant to cell. Ann Bot.

[54] Kosová K, Vítámvás P, Prásil IT, Renaut J (2011). Plant proteome changes under abiotic stress -- contribution of proteomics studies to understanding plant stress response. J Proteomics.

[55] Danshina PV, Schmalhausen EV, Avetisyan AV, Muronetz VI (2001). Mildly oxidized glyceraldehydes-3-phosphate dehydrogenase as a possible regulator of glycolysis. IUBMB Life.

[56] Bedhomme M, Adamo M, Marchand CH, Couturier J, Rouhier N, Lemaire SD (2012). Glutathionylation of cytosolic glyceraldehyde-3-phosphate dehydrogenase from the model plant *Arabidopsis thaliana* is reversed by both glutaredoxins and thioredoxins *in vitro*. Biochem J.

[57] Rochat T, Boudebbouze S, Gratadoux JJ, Blugeon S, Gaudu P, Langella P (2012). Proteomic analysis of spontaneous mutants of Lactococcus lactis: Involvement of GAPDH and arginine deiminase pathway in H_2_O_2_ resistance. Proteomics.

[58] Wierenga RK, Kapetaniou EG, Venkatesan R (2010). Triosephosphate isomerise: a highly evolved biocatalyst. Cell Mol Life Sci.

[59] Riccardi F, Gazeau P, de Vienne D, Zivy M (1998). Protein changes in response to progressive water deficit in maize: quantitative variation and polypeptide identification. Plant Physiol.

[60] Salekdeh GH, Siopongco J, Wade LJ, Ghareyazie B, Bennett J (2002). Proteomics analysis of rice leaves during drought stress and recovery. Proteomics.

[61] Umeda M, Hara C, Matsubayashi Y, Li HH, Liu Q, Tadokoro F (1994). Expressed sequence tags from cultured cells of rice (*Oryza sativa* L.) under stressed conditions: analysis of transcripts of genes engaged in ATP-generating pathways. Plant Mol Biol.

[62] Fei G, Zhou Y, Huang L, He D, Zhang G (2008). Proteomic analysis of long-term salinity stress-responsive proteins in *Thellungiella halophila* leaves. Chinese Sci Bull.

[63] Pandey S, Rai R, Rai LC (2012). Proteomics combines morphological, physiological and biochemical attributes to unravel the survival strategy of *Anabaena* sp. PCC7120 under arsenic stress. J Proteomics.

[64] Brugiere N, Dubois F, Limami AM, Lelandais M, Roux Y, Sangwan RS (1999). Glutamine synthetase in the phloem plays a major role in controlling proline production. Plant Cell.

[65] Gadaleta A, Nigro D, Giancaspro A, Blanco A (2011). The glutamine synthetase (GS2) genes in relation to grain protein content of durum wheat. Funct Integr Genomics.

[66] Díaz P, Betti M, Sánchez DH, Udvardi MK, Monza J, Márquez AJ (2010). Deficiency in plastidic glutamine synthetase alters proline metabolism and transcriptomic response in *Lotus japonicus* under drought stress. New Phytol.

[67] Roje S (2006). S-Adenosyl-L-methionine: beyond the universal methyl group donor. Phytochemistry.

[68] Roeder S, Dreschler K, Wirtz M, Cristescu SM, van Harren FJ, Hell R (2009). SAM levels, gene expression of SAM synthetase, methionine synthase and ACC oxidase, and ethylene emission from *N. suaveolens* flowers. Plant Mol Biol.

[69] Mayne MB, Coleman JR, Blumwald E (1996). Differential expression during drought conditioning of a root-specific S-adenosylmethionine synthetase from jack pine (*Pinus banksiana* Lamb.) seedlings. Plant Cell Environ.

[70] Quiroga M, Guerrero C, Botella MA, Ros Barceló A, Amaya I, Medina MI (2000). A tomato peroxidase involved in the synthesis of lignin and suberin. Plant Physiol.

[71] Sánchez-Aguayo I, Rodríguez-Galán JM, García R, Torreblanca J, Pardo JM (2004). Salt stress enhances xylem development and expression of S-adenosyl-l-methionine synthase in lignifying tissues of tomato plants. Planta.

[72] Yan XL, Liao H, Trull MC, Beebe SE, Lynch JP (2001). Induction of a major leaf acid phosphatase does not confer adaptation to low phosphorus availability in common bean. Plant Physiol.

[73] Yun SJ, Kaeppler SM (2001). Induction of maize acid phosphatase activities under phosphorus starvation. Plant Soil.

[74] Tian J, Liao H, Wang X, Cao A, Yan XL (2003). Phosphorus starvation-induced expression of leaf acid phosphatase isoforms in soybean. Acta Bot Sin.

[75] Tsuji H, Meguro N, Suzuki Y, Tsutsumi N, Hirai A, Nakazono M (2003). Induction of mitochondrial aldehyde dehydrogenase by submergence facilitates oxidation of acetaldehyde during re-aeration in rice. FEBS Lett.

[76] Marchitti SA, Brocker C, Stagos D, Vasiliou V (2008). Non-P450 aldehyde oxidizing enzymes: the aldehyde dehydrogenase superfamily. Expert Opin Drug Metab Toxicol.

[77] Sunkar R, Bartels D, Kirch HH (2003). Overexpression of a stress-inducible aldehyde dehydrogenase gene from *Arabidopsis thaliana* in transgenic plants improves stress tolerance. Plant J.

[78] Shiraishi T, Fukusaki E, Kobayashi A (2000). Formate dehydrogenase in rice plant: growth stimulation effect of formate in rice plant. J Biosci Bioeng.

[79] Alekseeva AA, Savin SS, Tishkov VI (2011). NAD(+)-dependent formate dehydrogenase from plants. Acta Nat.

[80] Bykova NV, Stensballe A, Egsgaard H, Jensen ON, Moller IM (2003). Phosphorylation of formate dehydrogenase in potato tuber mitochondria. J Biol Chem.

[81] Lehner I, Niehof M, Borlak J (2003). An optimized method for the isolation and identification of membrane proteins. Electrophoresis.

[82] Heide H, Kalisz HM, Follmann H (2004). The oxygen evolving enhancer protein 1 (OEE) of photosystem II in green algae exhibits thioredoxin activity. J Plant Physiol.

[83] Marin-Navarro J, Moreno J (2003). Modification of the proteolytic fragmentation pattern upon oxidation of cysteines from ribulose-1,5-bisphosphate carboxylase/oxygenase. Biochemistry.

[84] Flexas J, Ribas-Carbò M, Bota J, Galmés J, Henkle M, Martínez-Canellas S (2006). Decreased Rubisco activity during water stress is not induced by decreased relative water content but related to conditions of low stomatal conductance and chloroplast CO_2_ concentration. New Phytol.

[85] Galmés J, Aranjuelo I, Medrano H, Flexas J (2013). Variation in Rubisco content and activity under variable climatic factors. Photosynth Res.

[86] Nakahara K, Yamamoto H, Miyake C, Yokota A (2003). Purification and characterization of class-I and class-II fructose-1,6-bisphosphate aldolase from the cyanobacterium *Synechocystis* sp. PCC 6803. Plant Cell Physiol.

[87] Patron NJ, Rogers MB, Keeling PJ (2004). Gene replacement of fructose-1,6-bisphosphate aldolase supports the hypothesis of a single photosynthetic ancestor of chromalveolates. Eukaryot Cell.

[88] Yamazaki S, Nomata J, Fujita Y (2006). Differential operation of dual protochlorophyllide reductases for chlorophyll biosynthesis in response to environmental oxygen levels in the cyanobacterium *Leptolyngbya boryana*. Plant Physiol.

[89] Sakuraba Y, Rahman L, Cho SH, Kim YS, Koh HJ, Yoo SC (2013). The rice faded green leaf locus encodes protochlorophyllide oxidoreductase B and is essential for chlorophyll synthesis under high light conditions. Plant J.

[90] Reinbothe S, Reinbothe C (1996). The regulation of enzymes involved in chlorophyll biosynthesis. Eur J Biochem.

[91] Shepherd M, Reid JD, Hunter CN (2003). Purification and kinetic characterization of the magnesium protoporphyrin IX methyltransferase from *Synechocystis* PCC6803. Biochem J.

[92] Jiang GQ, Wang Z, Shang HH, Yang WL, Hu ZA, Phillips J (2007). Proteome analysis of leaves from the resurrection plant *Boea hygrometrica* in response to dehydration and rehydration. Planta.

[93] Frova C (2003). The plant glutathione transferase gene family: genomic structure, functions, expression and evolution. Physiol Plantarum.

[94] Frova C (2006). Glutathione transferases in the genomics era: New insights and perspectives. Biomol Eng.

[95] Margis R, Dunand C, Teixeira FK, Margis-Pinheiro M (2008). Glutathione peroxidase family: an evolutionary overview. J FEBS.

[96] Gill SS, Tuteja N (2010). Reactive oxygen species and antioxidant machinery in abiotic stress tolerance in crop plants. Plant Physiol Biochem.

[97] Suzuki N, Koussevitzky S, Mittler R, Miller G (2011). ROS and redox signaling in the response of plants to abiotic stress. Plant Cell Environ.

[98] Raven EL, Lad L, Sharp KH, Mewies M, Moody PC (2004). Defining substrate specificity and catalytic mechanism in ascorbate peroxidase. Biochem Soc Symp.

[99] Davletova S, Rizhsky L, Liang H, Shengqiang Z, Oliver DJ, Coutu J (2005). Cytosolic ascorbate peroxidase 1 is a central component of the reactive oxygen gene network of *Arabidopsis*. Plant Cell.

[100] Morgan MJ, Lehmann M, Schwarzländer M, Baxter CJ, Sienkiewicz-Porzucek A, Williams TC (2008). Decrease in manganese superoxide dismutase leads to reduced root growth and affects tricarboxylic acid cycle flux and mitochondrial redox homeostasis. Plant Physiol.

[101] Miriyala S, Spasojevic I, Tovmasyan A, Salvemini D, Vujaskovic Z, St Clair D (1822). Manganese superoxide dismutase, MnSOD and its mimics. Biochim Biophys Acta.

[102] Franklin CC, Backos DS, Mohar I, White CC, Forman HJ, Kavanagh TJ (2009). Structure, function, and post-translational regulation of the catalytic and modifier subunits of glutamate cysteine ligase. Mol Aspects Med.

[103] Lu SC (2009). Regulation of glutathione synthesis. Mol Aspects Med.

[104] Ganapathi S, Chidambaram P, Natarajan S, Vengoji R, Karuppannan V (2008). Combined expression of chitinase and β-1,3-glucanase genes in indica rice (*Oryza sativa* L.) enhances resistance against *Rhizoctonia solani*. Plant Sci.

[105] Meirinho S, Carvalho M, Dominguez Á, Choupina A (2010). Isolation and characterization by asymmetric PCR of the *ENDO1* gene for glucan endo-1,3-β-D-glucosidase in *Phytophthora cinnamomi* associated with the ink disease of *Castanea sativa* Mill. Braz Ach Bol Technol.

[106] Chen AP, Wang GL, Qu ZL, Lu CX, Liu N, Wang F (2007). Ectopic expression of ThCYP1, a stress-resposive cyclophilin gene from *Thellungiella halophila*, confers salt tolerance in fission yeast and tobacco cells. Plant Cell Rep.

[107] Gan PH, Shan W, Blackman LM, Hardham AR (2009). Characterization of cyclophilin-encoding genes in phytophthora. Mol Genet Genomics.

[108] Weng XY, Huang YY, Gao H, Sun JY (2010). Characterization of a xylanase inhibitor TAXI-I from wheat. Biol Plantarum.

[109] Moscetti I, Tundo S, Janni M, Sella L, Gazzetti K, Tauzin A (2013). Constitutive expression of the xylanase inhibitor TAXI-III delays Fusarium head blight symptoms in durum wheat transgenic plants. Mol Plant Microbe Interact.

[110] Hemant RK, Anil KS, Sudhir KS, Sneh LSP, Ashwani P (2009). Genome wide expression analysis of CBS domain containing proteins in *Arabidopsis thaliana* (L.) Heynh and *Oryza sativa* L. reveals their developmental and stress regulation. BMC Genomics.

[111] Singh AK, Kumar R, Pareek A, Sopory SK, Singla-Pareek SL (2012). Overexpression of rice CBS domain containing protein improves salinity, oxidative, and heavy metal tolerance in transgenic tobacco. Mol Biotechnol.

[112] Youssef A, Laizet Y, Block MA, Marechal E, Alcaraz JP, Larson TR (2009). Plant lipid-associated fibrillin proteins condition jasmonate production under photosynthetic stress. Plant J.

[113] Recklies AD, White C, Ling H (2002). The chitinase 3-like protein human cartilage glycoprotein 39 (HC-gp39) stimulates proliferation of human connective-tissue cells and activates both extracellular signal-regulated kinase- and protein kinase B-mediated signalling pathways. Biochem J.

[114] Liu JJ, Sturrock R, Ekramoddoullah AK (2010). The superfamily of thaumatin-like proteins: its origin, evolution, and expression towards biological function. Plant Cell Rep.

[115] Leyva JA, Bianchet MA, Amzel LM (2003). Understanding ATP synthesis: structure and mechanism of the F1-ATPase (Review). Mol Membr Biol.

[116] Itoh H, Takahashi A, Adachi K, Noji H, Yasuda R, Yoshida M (2004). Mechanically driven ATP synthesis by F1-ATPase. Nature.

[117] Christie AH, Allen GG, Gregory JT (2001). Induction of vacuolar ATPase and mitochondrial ATP synthase by aluminum in an aluminum-resistant cultivar of wheat. Plant Physiol.

[118] Zhang XX, Takano T, Liu SK (2006). Identification of a mitochondrial ATP synthase small subunit gene (*RMtATP6*) expressed in response to salts and osmotic stresses in rice (*Oryza sativa* L.). J Exp Bot.

[119] Zhang XX, Liu SK, Takano T (2008). Overexpression of a mitochondrial ATP synthase small subunit gene (*AtMtATP6*) confers tolerance to several abiotic stresses in *Saccharomyces cerevisiae* and *Arabidopsis thaliana*. Biotechnol Lett.

[120] Dzeja PP, Terzic A (2009). Adenylate kinase and AMP signaling networks: metabolic monitoring, signal communication and body energy sensing. Int J Mol Sci.

[121] Singh BN, Mishra RN, Agarwal PK, Goswami M, Nair S, Sopory SK (2004). A pea chloroplast translation elongation factor that is regulated by abiotic factors. Biochem Biophys Res Commun.

[122] Wan XY, Liu JY (2008). Comparative proteomics analysis reveals an intimate protein network provoked by hydrogen peroxide stress in rice seedling leaves. Mol Cell Proteomics.

[123] Ristic Z, Momcilović I, Fu J, Callegari E, DeRidder BP (2007). Chloroplast protein synthesis elongation factor, EF-Tu, reduces thermal aggregation of rubisco activase. J Plant Physiol.

[124] Margus T, Remm M, Tenson T (2011). A computational study of elongation factor G (EFG) duplicated genes: diverged nature underlying the innovation on the same structural template. PLoS One.

[125] Ma H, Song L, Shu Y, Wang S, Niu J, Wang Z (2012). Comparative proteomic analysis of seedling leaves of different salt tolerant soybean genotypes. J Proteomics.

[126] Rodnina M, Wintermeyer W (2011). The ribosome as a molecular machine: the mechanism of tRNA-mRNA movement in translocation. Biochem Soc Trans.

[127] Xu M, Wang Y, Chen L, Pan B, Chen F, Fang Y (2014). Down-regulation of ribosomal protein S15A mRNA with a short hairpin RNA inhibits human hepatic cancer cell growth *in vitro*. Gene.

[128] Palma JM, Sandalio LM, Corpas FJ, Romero-Puertas MC, McCarthy I, del Río LA (2002). Plant proteases, protein degradation, and oxidative stress: role of peroxisomes. Plant Physiol Bioch.

[129] Chamieh H, Marty V, Guetta D, Perollier A, Franzetti B (2012). Stress regulation of the PAN-proteasome system in the extreme halophilic archaeon *Halobacterium*. Extremophiles.

[130] Pang QY, Chen SX, Dai SJ, Chen YZ, Wang Y, Yan XF (2010). Comparative proteomics of salt tolerance in *Arabidopsis thaliana* and *Thellungiella halophila*. J Proteome Res.

[131] Wang W, Vinocur B, Shoseyov O, Altman A (2004). Role of plant heatshock proteins and molecular chaperones in the abiotic stress response. Trends Plant Sci.

[132] Feng JT, Liu YK, Song HY, Dai Z, Qin LX, Almofti MR (2005). Heat-shock protein 27: a potential biomarker for hepatocellular carcinoma identified by serum proteome analysis. Proteomics.

[133] Jiang Y, Yang B, Harris NS, Deyholos MK (2007). Comparative proteomics analysis of NaCl stress-responsive proteins in *Arabidopsis* roots. J Exp Bot.

[134] Cheng LX, Zhang X, Zhao QX, Li HJ, Wang YP, Wang DX (2014). Comparative proteomic analysis of cold-induced sweetening in potato (*Solanum tuberosum* L.) tuber. Acta Physiol Plant.

[135] Cuéllar J, Perales-Calvo J, Muga A, Valpuesta JM, Moro F (2013). Structural insights into the chaperone activity of the 40-kDa heat shock protein DnaJ: binding and remodeling of a native substrate. J Biol Chem.

[136] Maruyama T, Furuani M (2000). Archael Peptidyl-Prolyl cis/trans Isomerases (PPIases). Front Biosci.

[137] Pemberton TJ (2006). Identification and comparative analysis of sixteen fungal peptidyl-prolyl cis/trans isomerase repertoires. BMC Genomics.

[138] Hoogenboom BW, Suda K, Engel A, Fotiadis D (2007). The supramolecular assemblies of voltage-dependent anion channels in the native membrane. J Mol Biol.

[139] De Pinto V, Messina A, Lane DJR, Lawen A (2010). Voltage-dependent anion-selective channel (VDAC) in the plasma membrane. FEBS Lett.

[140] Wang L, He XL, Zhao YJ, Shen YZ, Huang ZJ (2011). Wheat vacuolar H^+^-ATPase subunit B cloning and its involvementin salt tolerance. Planta.

[141] Agarwal P, Reddy MK, Sopory SK, Agarwal PK (2009). Plant rabs: characterization, functional diversity, and role in stress tolerance. Plant Mol Biol Rep.

[142] Pollard TD (2007). Regulation of actin filament assembly by Arp2/3 complex and formins. Annu Rev Biophys Biomol Struct.

[143] Pollard TD, Cooper JA (2009). Actin, a central player in cell shape and movement. Science.

[144] Nakamura K, Zuppini A, Arnaudeau S, Lynch J, Ahsan I, Krause R (2001). Functional specialization of calreticulin domains. J Cell Biol.

[145] Chen MH, Tain GW, Gafni Y, Citovsky V (2005). Effects of calreticulin on viral cell-to-cell movement. Plant Physiol.

[146] Jia XY, Xu CY, Jing RL, Jing RL, Li RZ, Mao XG (2008). Molecular cloning and characterization of wheat calreticulin (CRT) gene involved in drought-stressed responses. J Exp Bot.

[147] Komatsu S, Yamada E, Furukawa K (2009). Cold stress changes the concanavalin A - positive glycosylation pattern of proteins expressed in the basal parts of rice leaf sheaths. Amino Acids.

[148] Pan YX, Wang XF, Liu HW, Zhang GY, Ma ZY (2010). Molecular cloning of three UDP-glucuronate decarboxylase genes that are preferentially expressed in *Gossypium* fibers from elongation to secondary cell wall synthesis. J Plant Biol.

[149] Yan J, Wang J, Zhang H (2002). An ankyrin repeat-containing protein plays a role in both disease resistance and antioxidation metabolism. Plant J.

[150] Wolpert TJ, Navarre DA, Moore DL, Macko V (1994). Identification of the 100-kD victorin binding protein from oats. Plant Cell.

[151] Liang L, Lai Z, Ma W, Zhang Y, Xue Y (2002). AhSL28, a senescence- and phosphate starvation-induced S-like RNase gene in *Antirrhinum*. Biochim Biophys Acta.

[152] Hugot K, Ponchet M, Marais A, Ricci P, Galiana E (2002). A tobacco S-like RNase inhibits hyphal elongation of plant pathogens. Mol Plant Microbe Interact.

[153] Zheng J, Wang YY, He YN, Zhou JJ, Li YP, Liu QQ (2014). Overexpression of an S-like ribonuclease gene, *OsRNS4*, confers enhanced tolerance to high salinity and hyposensitivity to phytochrome-mediated light signals in rice. Plant Sci.

[154] Zhou GA, Chang RZ, Qiu LJ (2010). Overexpression of soybean ubiquitin-conjugating enzyme gene *GmUBC2* confers enhanced drought and salt tolerance through modulating abiotic stress-responsive gene expression in *Arabidopsis*. Plant Mol Biol.

[155] Patridge EV, Ferry JG (2005). WrbA from *Escherichia coli* and *Archaeoglobus fulgidus* is an NAD(P)H: quinone oxidoreductase. J Bacteriol.

