# Supplementary figures and images for: Comparative proteomics illustrates the complexity of drought resistance mechanisms in two wheat (Triticum aestivum L.) cultivars under dehydration and rehydration
Source: BMC Plant Biol. 2016 Aug 31;16(1):188. doi: 10.1186/s12870-016-0871-8 (PMC5006382; doi:10.1186/s12870-016-0871-8)

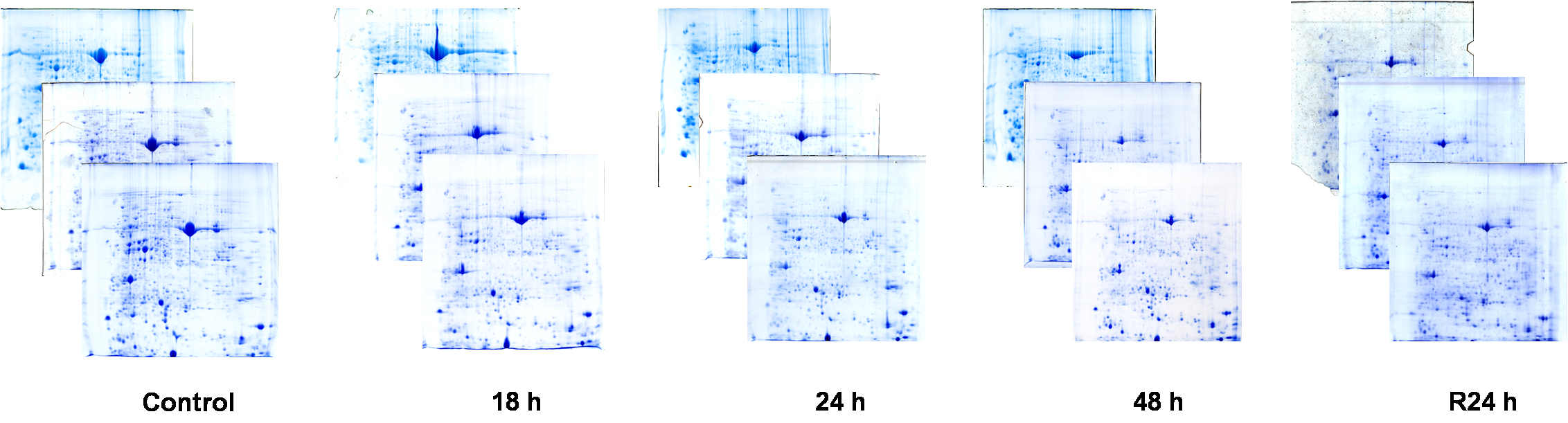

Supplement: Additional file 1: Figure S3. — The primary 2-DE gel maps at least three biological replicates for control, dehydration treatments (18 h, 24 h and 48 h) and rehydration treatment (R24 h) in Xihan No. 2. (TIF 1852 kb) [file 12870_2016_871_MOESM1_ESM.tif]

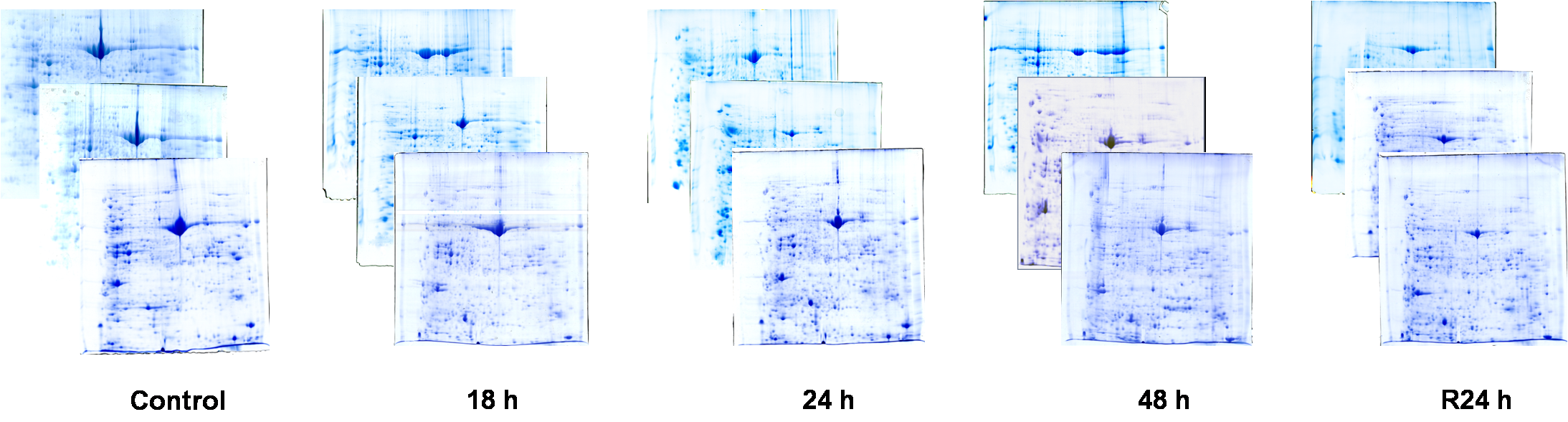

Supplement: Additional file 2: Figure S4. — The primary 2-DE gel maps at least three biological replicates for control, dehydration treatments (18 h, 24 h and 48 h) and rehydration treatment (R24 h) in Longchun 23. (TIF 1707 kb) [file 12870_2016_871_MOESM2_ESM.tif]

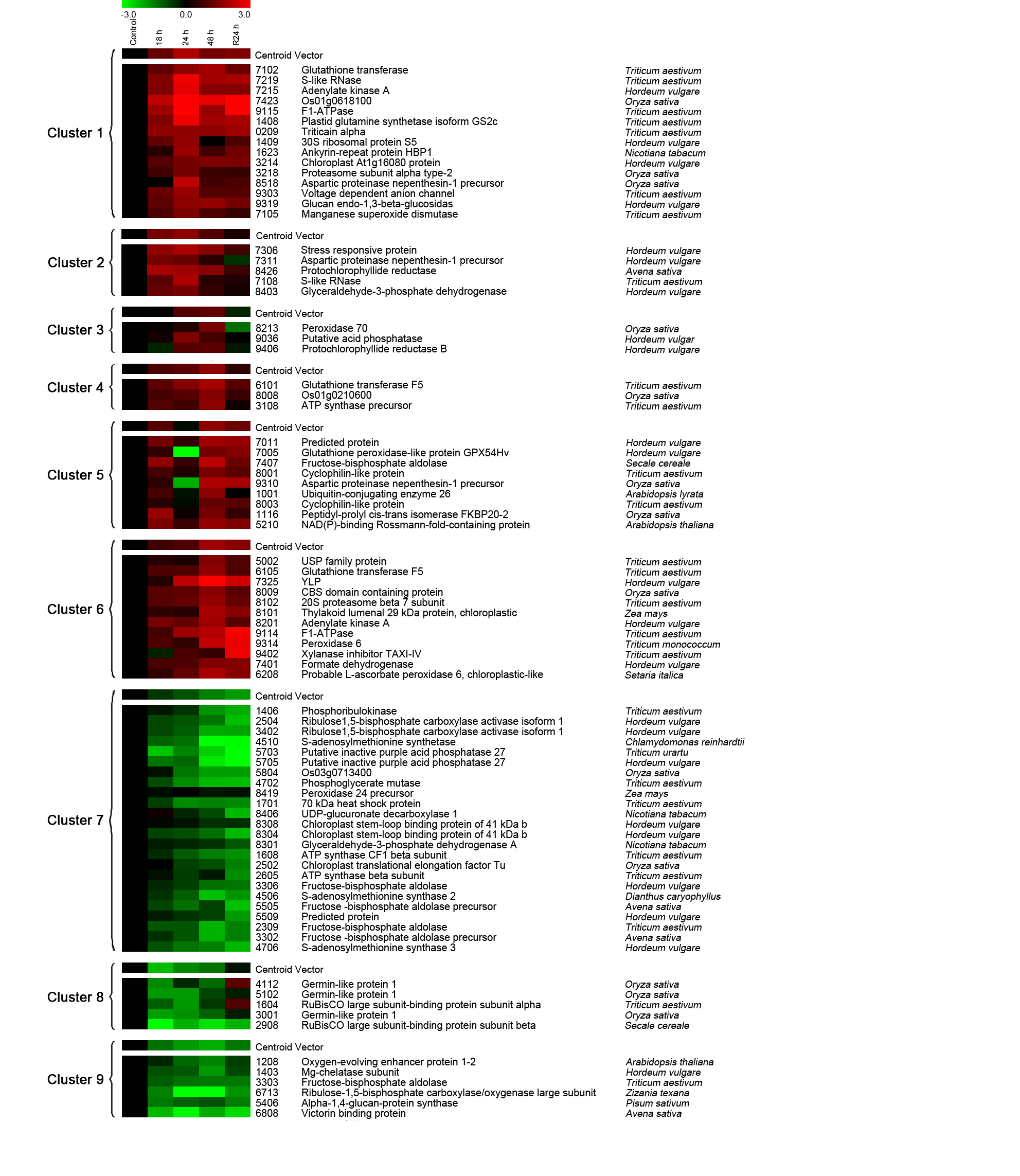

Supplement: Additional file 8: Figure S1. — The detailed information on differentially abundant proteins within each cluster in the clustering analysis of Xihan No. 2. The five columns of hierarchical cluster tree represent control, dehydration treatments (18 h, 24 h and 48 h) and rehydration treatment (R24 h), respectively. Each rows represent individual proteins. The up- and down-regulation of proteins are indicated in red and green, respectively. The intensity of colours is increased when the expression differences increased, as shown in the bar at the top. The differentially abundant proteins were grouped into 9 clusters in Xihan No. 2. The detailed information on these proteins within each cluster is presented, including the protein identification number, protein name and source organism. (TIF 13294 kb) [file 12870_2016_871_MOESM8_ESM.tif]

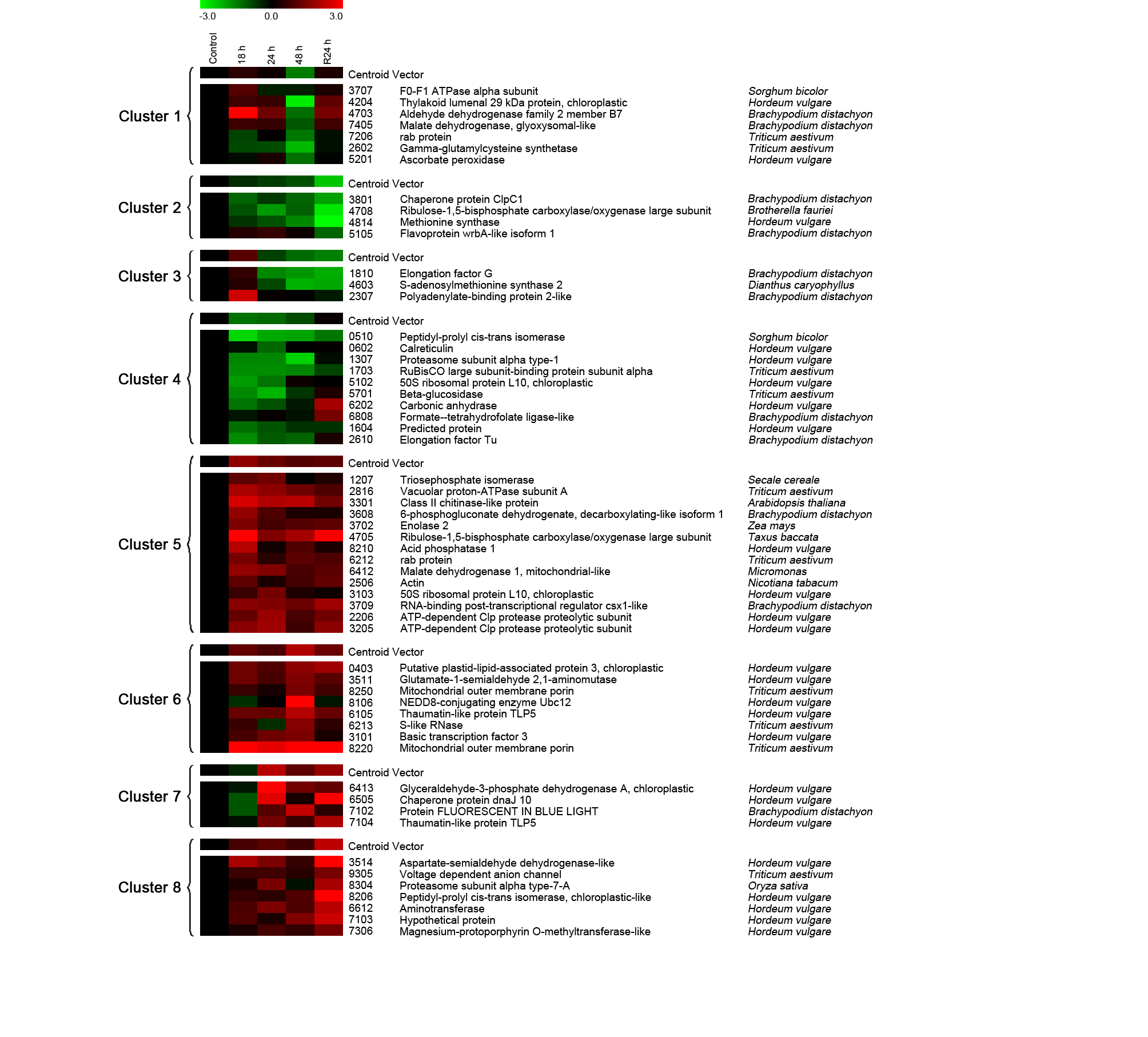

Supplement: Additional file 9: Figure S2. — The detailed information on differentially abundant proteins within each cluster in the clustering analysis of Longchun 23. The five columns of hierarchical cluster tree represent control, dehydration treatments (18 h, 24 h and 48 h) and rehydration treatment (R24 h), respectively. Each rows represent individual proteins. The up- and down-regulation of proteins are indicated in red and green, respectively. The intensity of colours is increased when the expression differences increased, as shown in the bar at the top. The differentially abundant proteins were grouped into 8 clusters in Longchun 23. The detailed information on these proteins within each cluster is presented, including the protein identification number, protein name and source organism. (TIF 10684 kb) [file 12870_2016_871_MOESM9_ESM.tif]
