# Supplementary material for: Comparative proteomics illustrates the complexity of drought resistance mechanisms in two wheat (Triticum aestivum L.) cultivars under dehydration and rehydration
Source: BMC Plant Biol. 2016 Aug 31;16(1):188. doi: 10.1186/s12870-016-0871-8 (PMC5006382; doi:10.1186/s12870-016-0871-8)
Supplement: Additional file 6: Table S4. — Identification of differentially abundant proteins associated with drought stress response in Xihan No. 2. Protein identifications were performed by searching for the Viridiplantae index of the NCBInr database using peptide mass fingerprinting (PMF) and MS/MS data from a MALDI-TOF/TOF mass spectrometry analysis. The spot number as given on the 2-DE gel image (shown in Fig. 3), the identified protein name, the source organism, the gene identification number as in GenBank, the number of matched peptides, the statistical score from the database, the sequence coverage (%), the theoretical Mass (kDa)/ pI values retrieved from protein database and the means for relative protein abundance ± standard error (SE) were listed. Spots with a significant differential expression are described as the spot volumes that were significantly different (p < 0.05, at least 2.5-fold) in relative abundance. (DOC 485 kb) [file 12870_2016_871_MOESM6_ESM.doc]

**Additional file 6: Table S4 Identification of differentially abundant proteins associated with drought stress response in Xihan No. 2.** Protein identifications were performed by searching for the *Viridiplantae* index of the NCBInr database using peptide mass fingerprinting (PMF) and MS/MS data from a MALDI-TOF/TOF mass spectrometry analysis. The spot number as given on the 2-DE gel image (shown in Fig. 3), the identified protein name, the source organism, the gene identification number as in GenBank, the number of matched peptides, the statistical score from the database, the sequence coverage (%), the theoretical Mass (kDa)/ pI values retrieved from protein database and the means for relative protein abundance ± standard error (SE) were listed. Spots with a significant differential expression are described as the spot volumes that were significantly different (*p*<0.05, at least 2.5-fold) in relative abundance.

| **Spot No.^a^** | **Protein Name** | **Organism** | **gi No.^b^** | **PN^c^** | **Score^d^** | **Coverage ^e^**  **(%)** | **Theoretical ^f^**  **pI/ Mr (Da)** | **Time Kinetics ^g^**  **0 18 24 48 R24** |
| --- | --- | --- | --- | --- | --- | --- | --- | --- |
| **Metabolism-related** **proteins** | | | | | | | | |
| TaL-8403 | Glyceraldehyde-3-phosphate dehydrogenase | *Hordeum vulgare* | gi|120680 | 6 | 361 | 24% | 6.67/36605 | 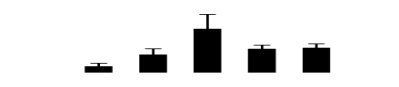 |
| TaL-8301 | Glyceraldehyde-3-phosphate dehydrogenase A | *Nicotiana tabacum* | gi|120661 | 2 | 83 | 6% | 6.60/42122 | 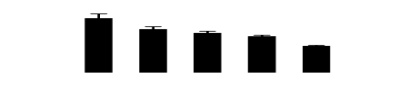 |
| TaL-4702 | Phosphoglycerate mutase | *Triticum aestivum* | gi|32400802 | 2 | 118 | 8% | 5.43/29615 | 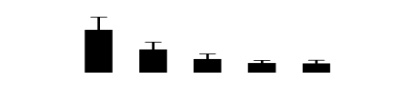 |
| TaL-9036 | Putative acid phosphatase | *Hordeum vulgar* | gi|41529149 | 1 | 84 | 4% | 8.62/29758 | 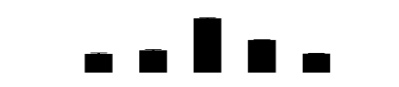 |
| TaL-9037 | Putative acid phosphatase | *Hordeum vulgare* | gi|41529149 | 1 | 65 | 4% | 8.62/29758 | 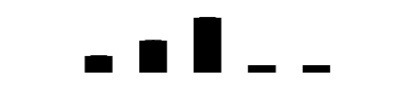 |
| TaL-5703 | Putative inactive purple acid phosphatase 27 | *Triticum urartu* | gi|326505368 | 5 | 188 | 9% | 6.21/69702 | 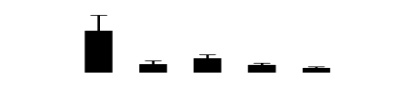 |
| TaL-5705 | Putative inactive purple acid phosphatase 27 | *Hordeum vulgare* | gi|326505368 | 6 | 342 | 12% | 6.21/69702 | 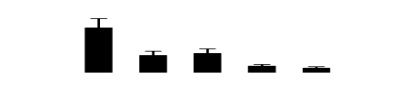 |
| TaL-7401 | Formate dehydrogenase | *Hordeum vulgare* | gi|21263612 | 3 | 171 | 9% | 6.90/41747 | 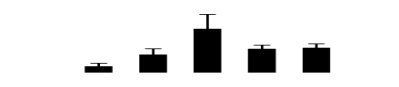 |
| TaL-1408 | Plastid glutamine synthetase isoform GS2c | *Triticum aestivum* | gi|71362640 | 6 | 604 | 21% | 5.75/47016 | 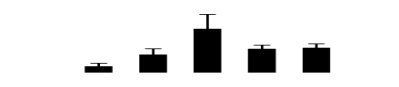 |
| TaL-4510 | S-adenosylmethionine synthase | *Chlamydomonas reinhardtii* | gi|2454484 | 4 | 240 | 21% | 9.47/19504 | 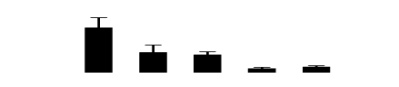 |
| TaL-4506 | S-adenosylmethionine synthase 2 | *Dianthus caryophyllus* | gi|127046 | 3 | 105 | 9% | 5.57/43618 | 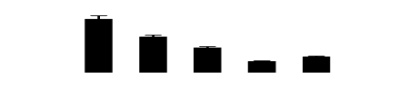 |
| TaL-4706 | S-adenosylmethionine synthase 3 | *Hordeum vulgare* | gi|122220777 | 5 | 415 | 17% | 5.51/43138 | 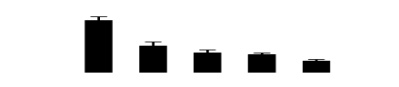 |
| **Photosynthesis-related proteins** | | | | | | | | |
| TaL-6713 | Ribulose-1,5-bisphosphate carboxylase/oxygenase large subunit | *Zizania texana* | gi|7240500 | 7 | 546 | 20% | 6.46/49381 | 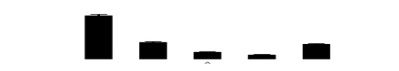 |
| TaL-2908 | RuBisCO large subunit-binding protein subunit beta | *Secale cereale* | gi|2493650 | 5 | 151 | 11% | 4.88/53721 | 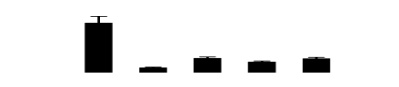 |
| TaL-1604 | RuBisCO large subunit-binding protein subunit alpha | *Triticum aestivum* | gi|134102 | 6 | 253 | 14% | 4.83/57656 | 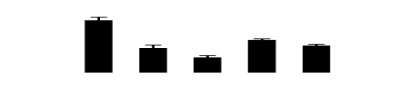 |
| TaL-3402 | Ribulose1,5-bisphosphate carboxylase activase isoform 1 | *Hordeum vulgare* | gi|167096 | 8 | 549 | 22% | 8.62/47341 | 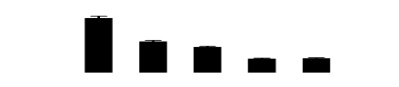 |
| TaL-2504 | Ribulose1,5-bisphosphate carboxylase activase isoform 1 | *Hordeum vulgare* | gi|167096 | 10 | 675 | 27% | 8.62/47341 | 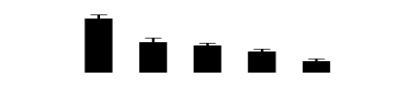 |
| TaL-1208 | Oxygen-evolving enhancer protein 1-2 | *Arabidopsis thaliana* | gi|15230324 | 3 | 208 | 13% | 5.92/35226 | 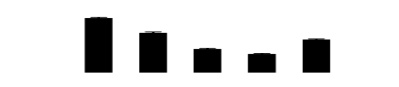 |
| TaL-7407 | Fructose-bisphosphate aldolase | *Secale cereale* | gi|226316439 | 4 | 170 | 13% | 6.39/39229 | 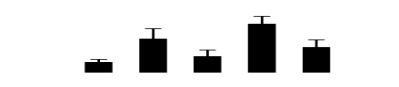 |
| TaL-2309 | Fructose-bisphosphate aldolase | *Triticum aestivum* | gi|223018643 | 6 | 456 | 23% | 5.94/42217 | 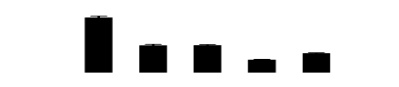 |
| TaL-3303 | Fructose-bisphosphate aldolase | *Triticum aestivum* | gi|223018643 | 3 | 101 | 12% | 5.94/42217 | 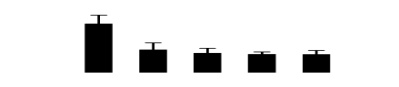 |
| TaL-3306 | Fructose-bisphosphate aldolase | *Hordeum vulgare* | gi|326499908 | 4 | 321 | 15% | 6.39/41753 | *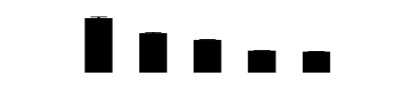* |
| TaL-3302 | Fructose -bisphosphate aldolase precursor | *Avena sativa* | gi|8272480 | 6 | 383 | 20% | 9.01/42124 | 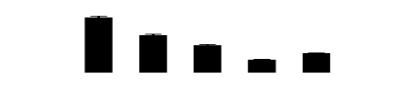 |
| TaL-5505 | Fructose -bisphosphate aldolase precursor | *Avena sativa* | gi|8272480 | 5 | 168 | 17% | 9.01/42124 | 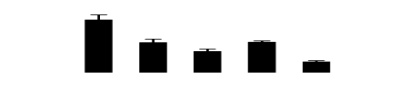 |
| TaL-8426 | Protochlorophyllide reductase | *Avena sativa* | gi|129707 | 3 | 78 | 8% | 7.81/34003 | 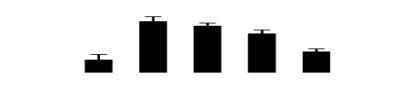 |
| TaL-9406 | Protochlorophyllide reductase B | *Hordeum vulgare* | gi|10720236 | 6 | 277 | 19% | 9.25/42350 | 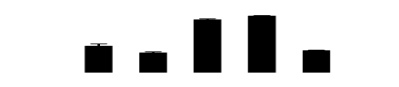 |
| TaL-1406 | Phosphoribulokinase | *Triticum aestivum* | gi|125580 | 6 | 282 | 15% | 5.72/45512 | 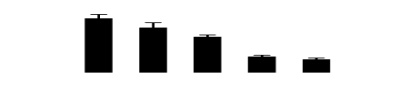 |
| TaL-1403 | Mg-chelatase subunit | *Hordeum vulgare* | gi|847873 | 2 | 149 | 8% | 4.89/36530 | 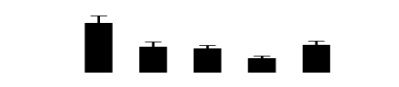 |
| TaL-3214 | Chloroplast At1g16080 protein | *Hordeum vulgare* | gi|326489475 | 2 | 161 | 9% | 6.45/32854 | 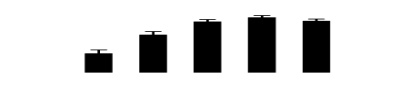 |
| TaL-8101 | Thylakoid lumenal 29 kDa protein, chloroplastic | *Zea mays* | gi|226531556 | 2 | 63 | 6% | 8.62/38684 | 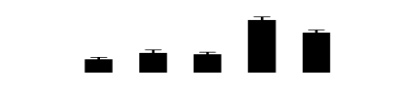 |
| **Redox homeostasis-related proteins** | | | | | | | | |
| TaL-7102 | Glutathione transferase | *Triticum aestivum* | gi|20067415 | 3 | 171 | 13% | 6.35/25098 | 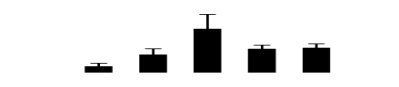 |
| TaL-6105 | Glutathione transferase F5 | *Triticum aestivum* | gi|23504745 | 3 | 96 | 24% | 5.78/23422 | 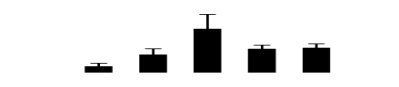 |
| TaL-6101 | Glutathione transferase F5 | *Triticum aestivum* | gi|23504745 | 4 | 218 | 29% | 5.78/23422 | 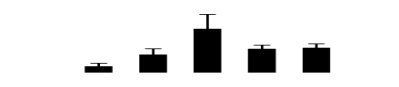 |
| TaL-7005 | Glutathione peroxidase-like protein GPX54Hv | *Hordeum vulgare* | gi|6179604 | 2 | 104 | 13% | 6.71/18418 | 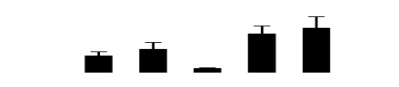 |
| TaL-9314 | Peroxidase 6 | *Triticum monococcum* | gi|57635157 | 2 | 99 | 11% | 8.51/35260 | 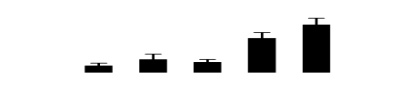 |
| TaL-8213 | Peroxidase 70 | *Oryza sativa* | gi|125538753 | 1 | 49 | 3% | 7.51/34747 | 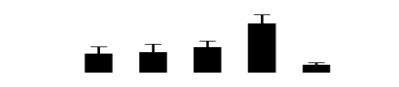 |
| TaL-8419 | Peroxidase 24 precursor | *Zea mays* | gi|195605894 | 1 | 76 | 4% | 8.81/37038 | 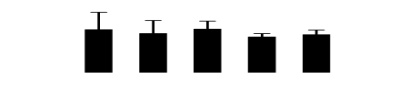 |
| TaL-6208 | Probable L-ascorbate peroxidase 6, chloroplastic-like | *Setaria italica* | gi|223947673 | 3 | 136 | 12% | 7.75/34284 | 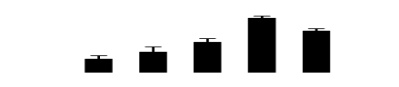 |
| TaL-7105 | Manganese superoxide dismutase | *Triticum aestivum* | gi|1654387 | 5 | 273 | 29% | 7.89/25283 | 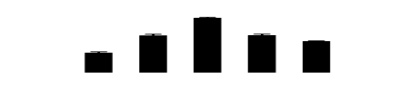 |
| **Defence-related proteins** | | | | | | | | |
| TaL-9319 | Glucan endo-1,3-beta-glucosidase | *Hordeum vulgare* | gi|167051 | 3 | 141 | 16% | 8.46/32678 | 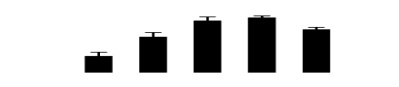 |
| TaL-8001 | Cyclophilin-like protein | *Triticum aestivum* | gi|37788308 | 3 | 169 | 17% | 9.40/26062 | 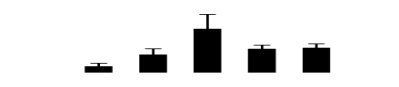 |
| TaL-8003 | Cyclophilin-like protein | *Triticum aestivum* | gi|37788308 | 5 | 335 | 24% | 9.40/26062 | 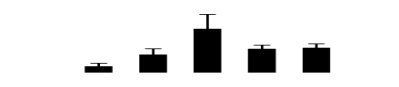 |
| TaL-8009 | CBS domain containing protein | *Oryza sativa* | gi|108710960 | 1 | 66 | 5% | 8.83/25095 | 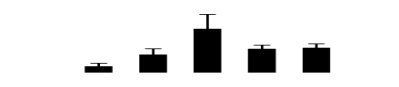 |
| TaL-5210 | NAD(P)-binding Rossmann-fold-containing protein | *Arabidopsis thaliana* | gi|18404496 | 1 | 132 | 5% | 8.37/34972 | 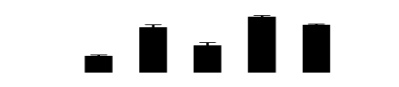 |
| TaL-5406 | Alpha-1,4-glucan-protein synthase | *Pisum sativum* | gi|34582497 | 4 | 177 | 8%/ | 5.73/42059 | 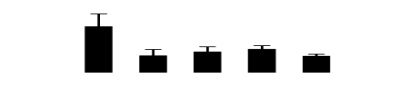 |
| TaL-5102 | Germin-like protein 1 | *Oryza sativa* | gi|4239821 | 1 | 96 | 10% | 6.01/22017 | 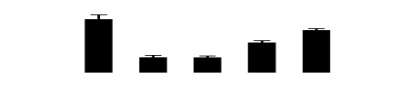 |
| TaL-4112 | Germin-like protein 1 | *Oryza sativa* | gi|4239821 | 1 | 102 | 10% | 6.01/22017 | 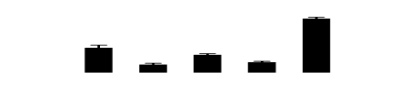 |
| TaL-3001 | Germin-like protein 1 | *Oryza sativa* | gi|4239821 | 1 | 78 | 10% | 6.01/22017 | 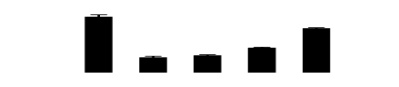 |
| TaL-9402 | Xylanase inhibitor TAXI-IV | *Triticum aestivum* | gi|56201272 | 1 | 46 | 2% | 8.66/42687 | 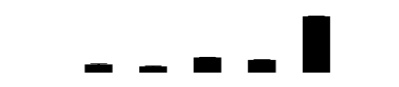 |
| TaL-7306 | Stress responsive protein | *Hordeum vulgare* | gi|326497973 | 3 | 189 | 10% | 6.27/39056 | 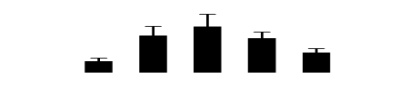 |
| TaL-5002 | USP family protein | *Triticum aestivum* | gi|60100214 | 2 | 97 | 15% | 5.78/17967 | 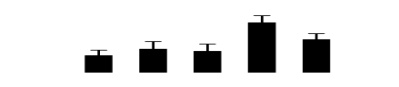 |
| **Energy-related proteins** | | | | | | | | |
| TaL-3108 | ATP synthase precursor | *Triticum aestivum* | gi|47607439 | 2 | 114 | 7% | 7.71/27090 | 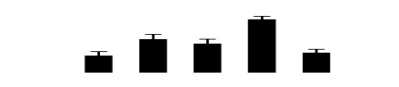 |
| TaL-9114 | F1-ATPase | *Triticum aestivum* | gi|32400863 | 2 | 66 | 16% | 9.99/19950 | 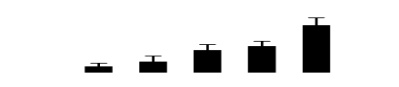 |
| TaL-9115 | F1-ATPase | *Triticum aestivum* | gi|32400863 | 5 | 137 | 21% | 9.99/19950 | 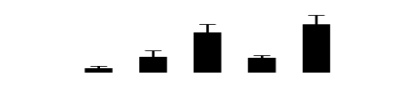 |
| TaL-1608 | ATP synthase CF1 beta subunit | *Triticum aestivum* | gi|14017579 | 8 | 657 | 25% | 5.06/53881 | 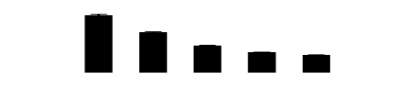 |
| TaL-2605 | ATP synthase beta subunit | *Triticum aestivum* | gi|525291 | 7 | 557 | 18% | 5.56/59326 | 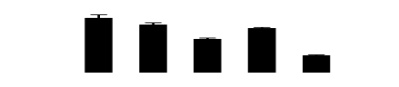 |
| TaL-8201 | Adenylate kinase A | *Hordeum vulgare* | gi|326496737 | 4 | 161 | 17% | 7.66/26477 | 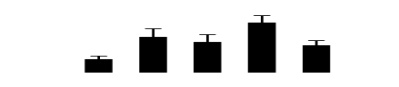 |
| TaL-7215 | Adenylate kinase A | *Hordeum vulgare* | gi|326517593 | 6 | 190 | 26% | 6.54/26642 | 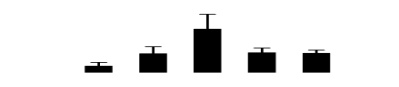 |
| **Protein translation, processing and degradation-related proteins** | | | | | | | | |
| TaL-2502 | Chloroplast translational elongation factor Tu | *Oryza sativa* | gi|6525065 | 4 | 154 | 12% | 6.05/50551 | 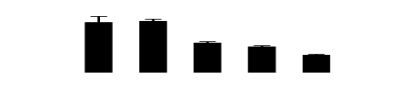 |
| TaL-1409 | 30S ribosomal protein S5 | *Hordeum vulgare* | gi|326506396 | 5 | 142 | 13% | 5.08/35439 | 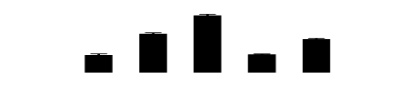 |
| TaL-1116 | Peptidyl-prolyl cis-trans isomerase FKBP20-2 | *Oryza sativa* | gi|115472151 | 3 | 84 | 12% | 9.49/28236 | 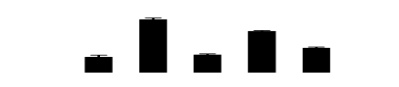 |
| TaL-3218 | Proteasome subunit alpha type-2 | *Oryza sativa* | gi|115447473 | 4 | 181 | 17% | 5.39/25828 | 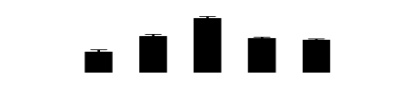 |
| TaL-8102 | 20S proteasome beta 7 subunit | *Triticum aestivum* | gi|52548242 | 4 | 124 | 27% | 6.91/23801 | 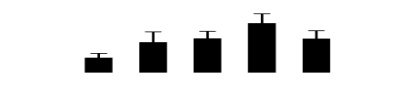 |
| TaL-8518 | Aspartic proteinase nepenthesin-1 precursor | *Oryza sativa* | gi|115451209 | 2 | 78 | 4% | 6.64/45733 | 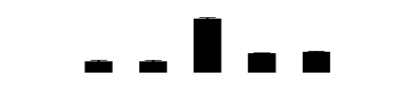 |
| TaL-9310 | Aspartic proteinase nepenthesin-1 precursor | *Oryza sativa* | gi|115451209 | 3 | 140 | 6% | 6.64/45733 | 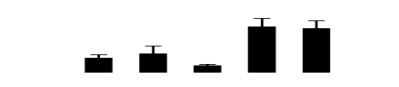 |
| TaL-7311 | Aspartic proteinase nepenthesin-1 precursor | *Hordeum vulgare* | gi|326528187 | 2 | 53 | 6% | 8.37/34405 | 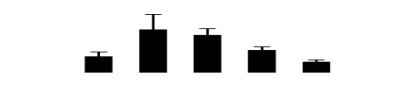 |
| TaL-0209 | Triticain alpha | *Triticum aestivum* | gi|111073715 | 1 | 86 | 3% | 5.01/51572 | 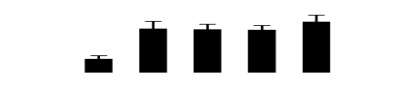 |
| TaL-1701 | 70 kDa heat shock protein | *Triticum aestivum* | gi|254211611 | 3 | 131 | 6% | 5.01/73724 | 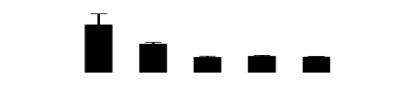 |
| TaL-2701 | Chloroplast envelope membrane  70 kDa heat shock-related protein | *Spinacia oleracea* | gi|123656 | 2 | 99 | 4% | 5.34/72143 | 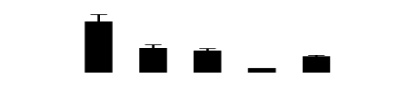 |
| **Transcription-related proteins** | | | | | | | | |
| TaL-8304 | Chloroplast stem-loop binding protein of 41 kDa b | *Hordeum vulgare* | gi|326531332 | 7 | 238 | 23% | 8.87/41017 | 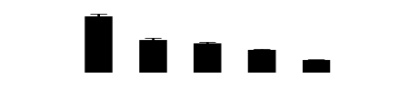 |
| TaL-8308 | Chloroplast stem-loop binding protein of 41 kDa b | *Hordeum vulgare* | gi|326531332 | 7 | 465 | 23% | 8.87/41017 | 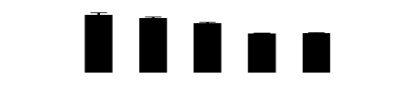 |
| **Transport-related proteins** | | | | | | | | |
| TaL-9303 | Voltage dependent anion channel | *Triticum aestivum* | gi|558650 | 3 | 62 | 15% | 9.33/29298 | 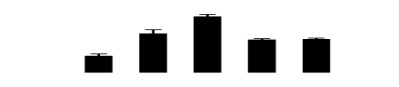 |
| TaL-7325 | YLP | *Hordeum vulgare* | gi|4099148 | 6 | 289 | 20% | 6.57/26359 | 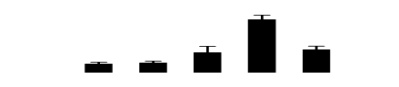 |
| **Miscellaneous** | | | | | | | | |
| TaL-8406 | UDP-glucuronate decarboxylase 1 | *Nicotiana tabacum* | gi|48093461 | 4 | 97 | 9% | 7.10/38875 | 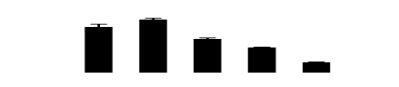 |
| TaL-7219 | S-like RNase | *Triticum aestivum* | gi|20271131 | 1 | 57 | 7% | 6.30/28320 | 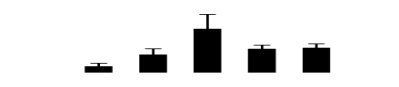 |
| TaL-7108 | S-like RNase | *Triticum aestivum* | gi|20271131 | 5 | 421 | 23% | 6.30/28320 | 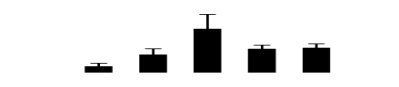 |
| TaL-1001 | Ubiquitin-conjugating enzyme 26 | *Arabidopsis lyrata* | gi|297795885 | 5 | 76 | 18% | 4.90/21425 | 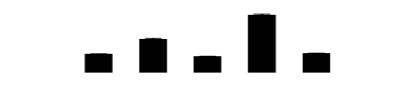 |
| TaL-1623 | Ankyrin-repeat protein HBP1 | *Nicotiana tabacum* | gi|13310811 | 1 | 53 | 3% | 4.45/37522 | 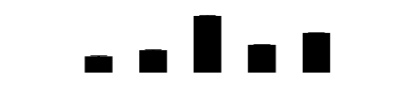 |
| TaL-6808 | Victorin binding protein | *Avena sativa* | gi|710308 | 4 | 95 | 6% | 6.48/112298 | 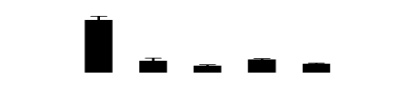 |
| **Unknown** | | | | | | | | |
| TaL-8008 | Os01g0210600 | *Oryza sativa* | gi|115435222 | 1 | 51 | 4% | 8.61/18196 | 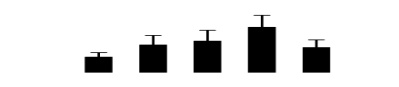 |
| TaL-5804 | Os03g0713400 | *Oryza sativa* | gi|115454943 | 3 | 65 | 4% | 5.86/82148 | 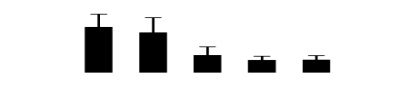 |
| TaL-7423 | Os01g0618100 | *Oryza sativa* | gi|115438617 | 3 | 175 | 11% | 6.28/28234 | 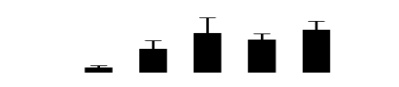 |
| TaL-7011 | Predicted protein | *Hordeum vulgare* | gi|326494242 | 3 | 212 | 23% | 9.07/23292 | 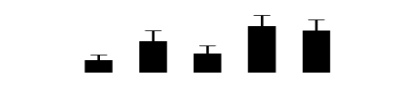 |
| TaL-5509 | Predicted protein | *Hordeum vulgare* | gi|326505840 | 5 | 225 | 11% | 7.88/52587 | 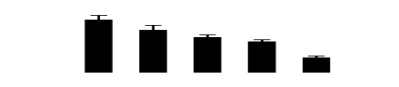 |

^a^ Spot No. corresponds to position of the spot in the gel as illustrated in Fig. 3.

^b^ Gene identification number as in GenBank.

^c^ The number of matched peptides.

^d^ The score is calculated with MASCOT. Ions score is -10*Log (P), where P is the probability that the observed match is a random event.

^e^ The sequence coverage percentage (%).

^f^ Theoretical *pI* and mass (Da) values of identified proteins estimated with MASCOT.

^g^ Time kinetics represents the average change of spot abundance at various time points 0, 18, 24, 48, R24 h (rehydration treatment). The data were taken in terms of -fold expression with respect to the control value and were log-transformed to the base two in order to level the scale of expression and to reduce the noise.
