# Supplementary material for: Comparative proteomics illustrates the complexity of drought resistance mechanisms in two wheat (Triticum aestivum L.) cultivars under dehydration and rehydration
Source: BMC Plant Biol. 2016 Aug 31;16(1):188. doi: 10.1186/s12870-016-0871-8 (PMC5006382; doi:10.1186/s12870-016-0871-8)
Supplement: Additional file 7: Table S5. — Identification of differentially abundant proteins associated with drought stress response in Longchun 23. Protein identifications were performed by searching for the Viridiplantae index of the NCBInr database using peptide mass fingerprinting (PMF) and MS/MS data from a MALDI-TOF/TOF mass spectrometry analysis. The spot number as given on the 2-DE gel image (shown in Fig. 4), the identified protein name, the source organism, the gene identification number as in GenBank, the number of matched peptides, the statistical score from the database, the sequence coverage (%), the theoretical Mass (kDa)/pI values retrieved from protein database and the means for relative protein abundance ± standard error (SE) were listed. Spots with a significant differential expression are described as the spot volumes that were significantly different (p < 0.05, at least 2.5-fold) in relative abundance. (DOC 372 kb) [file 12870_2016_871_MOESM7_ESM.doc]

**Additional file 7: Table S5 Identification of differentially abundant proteins associated with drought stress response in Longchun 23.** Protein identifications were performed by searching for the *Viridiplantae* index of the NCBInr database using peptide mass fingerprinting (PMF) and MS/MS data from a MALDI-TOF/TOF mass spectrometry analysis. The spot number as given on the 2-DE gel image (shown in Fig. 4), the identified protein name, the source organism, the gene identification number as in GenBank, the number of matched peptides, the statistical score from the database, the sequence coverage (%), the theoretical Mass (kDa)/ pI values retrieved from protein database and the means for relative protein abundance ± standard error (SE) were listed. Spots with a significant differential expression are described as the spot volumes that were significantly different (*p*<0.05, at least 2.5-fold) in relative abundance.

| **Spot No.^a^** | **Protein Name** | **Organism** | **gi No.^b^** | **PN^c^** | **Score^d^** | **Coverage ^e^**  **(%)** | **Theoretical ^f^**  **pI/ Mr (Da)** | **Time Kinetics ^g^**  **0 18 24 48 R24** |
| --- | --- | --- | --- | --- | --- | --- | --- | --- |
| **Metabolism-related** **proteins** | | | | | | | | |
| TaL-3608 | 6-phosphogluconate dehydrogenate, decarboxylating-like isoform 1 | *Brachypodium distachyon* | gi|357110692 | 7 | 371 | 20% | 5.61/52860 | 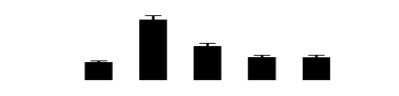 |
| TaL-3806 | Transketolase, chloroplastic | *Hordeum vulgare* | gi|326533372 | 8 | 255 | 11% | 5.45/74032 | 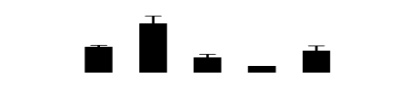 |
| TaL-6413 | Glyceraldehyde-3-phosphate dehydrogenase A, chloroplastic | *Hordeum vulgare* | gi|326500100 | 4 | 137 | 12% | 7.60/43015 | 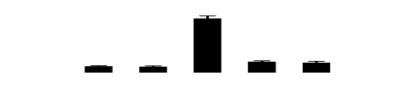 |
| TaL-1207 | Triosephosphate isomerase | *Secale cereale* | gi|1174745 | 3 | 145 | 13% | 6.00/31955 | 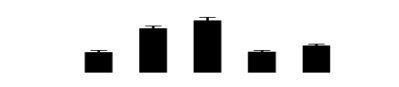 |
| TaL-3702 | Enolase 2 | *Zea mays* | gi|162460735 | 3 | 162 | 9% | 5.70/48418 | 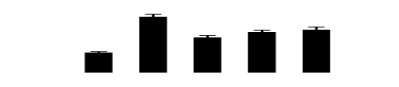 |
| TaL-8210 | Acid phosphatase 1 | *Hordeum vulgare* | gi|326489953 | 2 | 98 | 9% | 9.22/29881 | 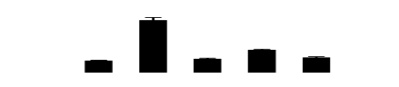 |
| TaL-6412 | Malate dehydrogenase 1, mitochondrial-like | *Micromonas* | gi|255073915 | 2 | 186 | 3% | 4.95/33823 | 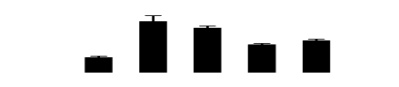 |
| TaL-7405 | Malate dehydrogenase, glyoxysomal-like | *Brachypodium distachyon* | gi|357113800 | 6 | 337 | 21% | 8.39/37990 | 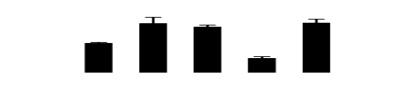 |
| TaL-4703 | Aldehyde dehydrogenase family 2 member B7 | *Brachypodium distachyon* | gi|357124561 | 4 | 209 | 6% | 7.17/59436 | 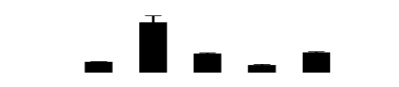 |
| TaL-6808 | Formate--tetrahydrofolate ligase-like | *Brachypodium distachyon* | gi|357153847 | 2 | 46 | 2% | 8.16/77679 | 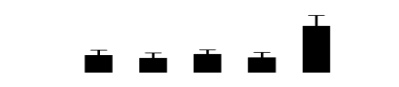 |
| TaL-3500 | Putative 1-deoxy-D-xylulose 5-phosphate reductoisomerase | *Hordeum vulgare* | gi|34996397 | 5 | 290 | 11% | 6.44/52997 | 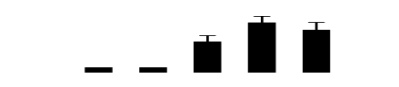 |
| TaL-4814 | Methionine synthase | *Hordeum vulgare* | gi|50897038 | 5 | 240 | 10% | 5.68/84794 | 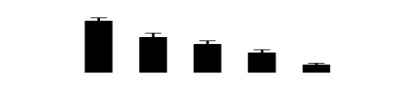 |
| TaL-4603 | S-adenosylmethionine synthase 2 | *Dianthus caryophyllus* | gi|127046 | 3 | 103 | 7% | 5.57/43618 | 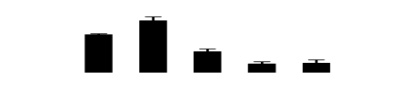 |
| TaL-3514 | Aspartate-semialdehyde dehydrogenase-like | *Hordeum vulgare* | gi|326500176 | 2 | 118 | 10% | 6.04/40573 | 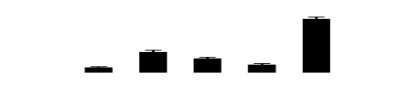 |
| TaL-6612 | Aminotransferase | *Hordeum vulgare* | gi|326487678 | 5 | 188 | 12% | 6.90/55806 | 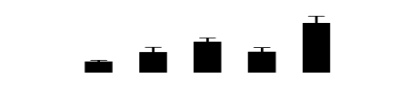 |
| **Photosynthesis-related proteins** | | | | | | | | |
| TaL-4708 | Ribulose-1,5-bisphosphate carboxylase/oxygenase large subunit | *Brotherella fauriei* | gi|9967379 | 2 | 73 | 4% | 6.18/51626 | 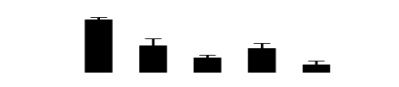 |
| TaL-4705 | Ribulose-1,5-bisphosphate carboxylase/oxygenase large subunit | *Taxus baccata* | gi|21360798 | 3 | 176 | 9% | 6.91/49254 | 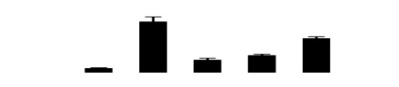 |
| TaL-6214 | rbcL | *Triticum aestivum* | gi|12344 | 2 | 95 | 5% | 6.60/47600 | 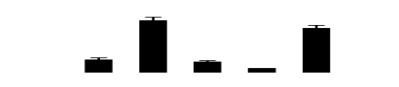 |
| TaL-1703 | RuBisCO large subunit-binding protein subunit alpha | *Triticum aestivum* | gi|134102 | 7 | 474 | 15% | 4.83/57656 | 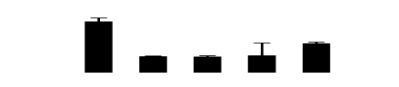 |
| TaL-4113 | Oxygen-evolving enhancer protein 2 | *Triticum aestivum* | gi|131394 | 7 | 366 | 33% | 8.84/27424 | 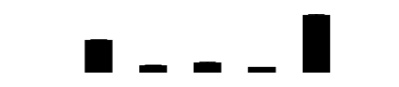 |
| TaL-4204 | Thylakoid lumenal 29 kDa protein, chloroplastic | *Hordeum vulgare* | gi|326513514 | 5 | 274 | 16% | 7.57/37707 | 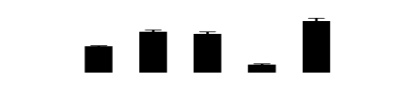 |
| TaL-3508 | Glutamate-1-semialdehyde 2,1-aminomutase | *Hordeum vulgare* | gi|1170029 | 8 | 300 | 22% | 6.39/49690 | 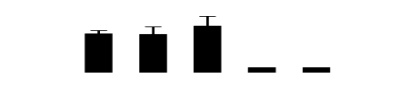 |
| TaL-3511 | Glutamate-1-semialdehyde 2,1-aminomutase | *Hordeum vulgare* | gi|1170029 | 8 | 351 | 22% | 6.39/49690 | 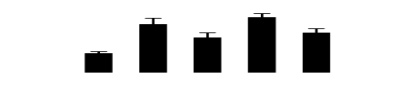 |
| TaL-6202 | Carbonic anhydrase | *Hordeum vulgare* | gi|729003 | 5 | 128 | 20% | 8.93/35736 | 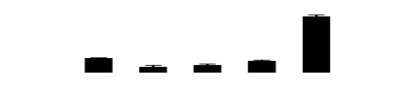 |
| TaL-7306 | Magnesium-protoporphyrin O-methyltransferase-like | *Hordeum vulgare* | gi|326492700 | 7 | 347 | 21% | 8.73/34889 | 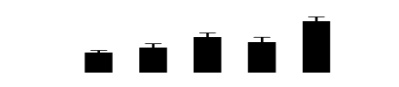 |
| **Redox homeostasis-related proteins** | | | | | | | | |
| TaL-2602 | Gamma-glutamylcysteine synthetase | *Triticum aestivum* | gi|57903694 | 4 | 185 | 12% | 5.30/43079 | 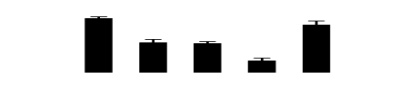 |
| TaL-5201 | Ascorbate peroxidase | *Hordeum vulgare* | gi|3688398 | 7 | 453 | 34% | 5.85/27532 | 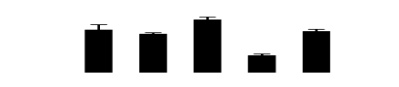 |
| **Defence-related proteins** | | | | | | | | |
| TaL-5701 | Beta-glucosidase | *Triticum aestivum* | gi|90990912 | 5 | 202 | 11% | 6.55/64980 | 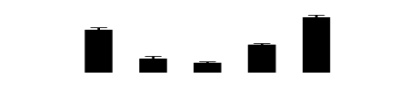 |
| TaL-7104 | Thaumatin-like protein TLP5 | *Hordeum vulgare* | gi|56682582 | 3 | 122 | 18% | 6.04/25813 | 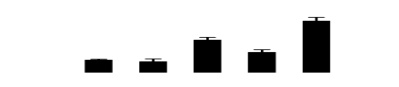 |
| TaL-6105 | Thaumatin-like protein TLP5 | *Hordeum vulgare* | gi|56682582 | 3 | 140 | 18% | 6.04/25813 | 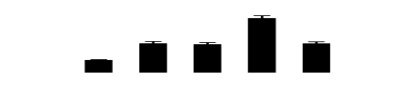 |
| TaL-3301 | Class II chitinase-like protein | *Arabidopsis thaliana* | gi|15234281 | 1 | 54 | 5% | 9.04/31901 | 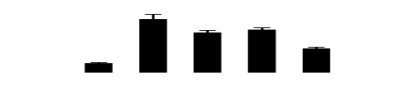 |
| TaL-0403 | Putative plastid-lipid-associated protein 3, chloroplastic | *Hordeum vulgare* | gi|326491997 | 4 | 243 | 16% | 4.74/39570 | 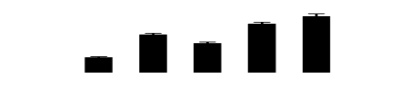 |
| **Energy-related proteins** | | | | | | | | |
| TaL-3707 | F0-F1 ATPase alpha subunit | *Sorghum bicolor* | gi|9408184 | 6 | 275 | 19% | 5.96/47840 | 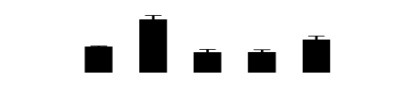 |
| **Protein translation, processing and degradation-related proteins** | | | | | | | | |
| TaL-1810 | Elongation factor G | *Brachypodium distachyon* | gi|357164996 | 8 | 299 | 16% | 5.25/84279 | 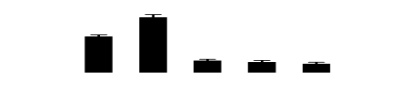 |
| TaL-2610 | Elongation factor Tu | *Brachypodium distachyon* | gi|357149925 | 7 | 428 | 19% | 5.88/50638 | 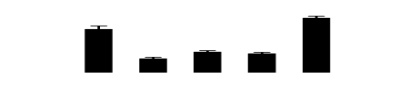 |
| TaL-2307 | Polyadenylate-binding protein 2-like | *Brachypodium distachyon* | gi|357124786 | 4 | 188 | 32% | 5.28/23606 | 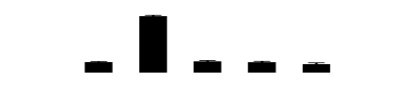 |
| TaL-3103 | 50S ribosomal protein L10, chloroplastic | *Hordeum vulgare* | gi|326507838 | 5 | 160 | 34% | 8.31/24197 | 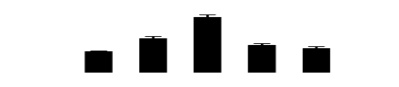 |
| TaL-5102 | 50S ribosomal protein L10, chloroplastic | *Hordeum vulgare* | gi|326507838 | 3 | 166 | 14% | 8.31/24197 | 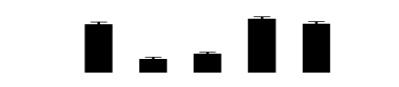 |
| TaL-8206 | Peptidyl-prolyl cis-trans isomerase, chloroplastic-like | *Hordeum vulgare* | gi|326499938 | 6 | 335 | 24% | 9.67/30263 | 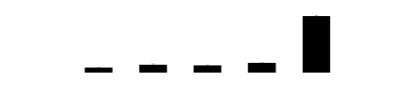 |
| TaL-0510 | Peptidyl-prolyl cis-trans isomerase | *Sorghum bicolor* | gi|242079005 | 3 | 84 | 5% | 4.83/46694 | 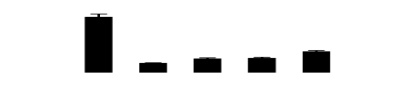 |
| TaL-8304 | Proteasome subunit alpha type-7-A | *Oryza sativa* | gi|115477683 | 6 | 208 | 26% | 6.93/27393 | 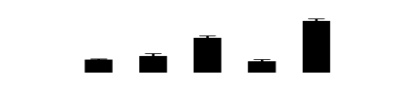 |
| TaL-1307 | Proteasome subunit alpha type-1 | *Hordeum vulgare* | gi|326506152 | 4 | 283 | 20% | 5.15/31518 | 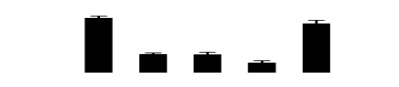 |
| TaL-3205 | ATP-dependent Clp protease proteolytic subunit | *Hordeum vulgare* | gi|326492708 | 5 | 114 | 23% | 5.64/30850 | 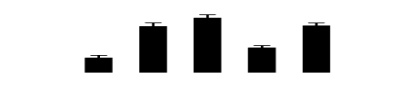 |
| TaL-2206 | ATP-dependent Clp protease proteolytic subunit | *Hordeum vulgare* | gi|326492708 | 5 | 147 | 23% | 5.63/30850 | 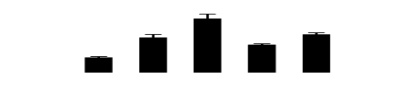 |
| TaL-6505 | Chaperone protein dnaJ 10 | *Hordeum vulgare* | gi|326517587 | 3 | 68 | 15% | 6.63/35240 | 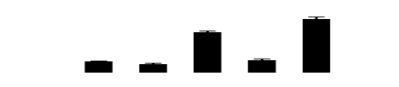 |
| TaL-3801 | Chaperone protein ClpC1 | *Brachypodium distachyon* | gi|357149201 | 8 | 373 | 14% | 5.95/97690 | 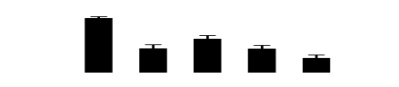 |
| **Transcription-related proteins** | | | | | | | | |
| TaL-3709 | RNA-binding post-transcriptional regulator csx1-like | *Brachypodium distachyon* | gi|326513006 | 3 | 102 | 7% | 5.79/47389 | 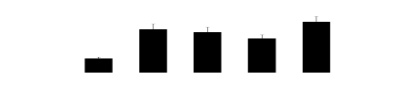 |
| TaL-3101 | Basic transcription factor 3 | *Hordeum vulgare* | gi|326504174 | 4 | 190 | 32% | 5.99/19192 | 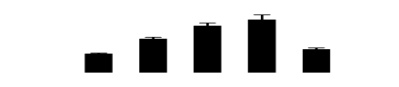 |
| **Transport-related proteins** | | | | | | | | |
| TaL-2816 | Vacuolar proton-ATPase subunit A | *Triticum aestivum* | gi|90025017 | 7 | 294 | 13% | 5.23/68754 | 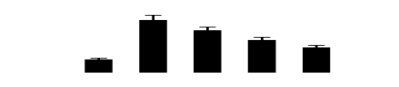 |
| TaL-9305 | Voltage dependent anion channel | *Triticum aestivum* | gi|558650 | 6 | 348 | 35% | 9.33/29298 | 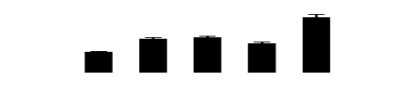 |
| TaL-6212 | rab protein | *Triticum aestivum* | gi|21853 | 3 | 191 | 14% | 9.22/23215 | 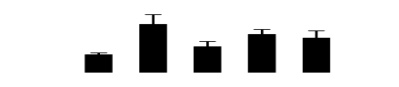 |
| TaL-6215 | rab protein | *Triticum aestivum* | gi|21853 | 2 | 126 | 13% | 9.22/23215 | 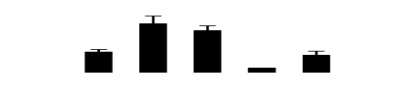 |
| TaL-7206 | rab protein | *Triticum aestivum* | gi|21853 | 3 | 111 | 14% | 9.22/23215 | 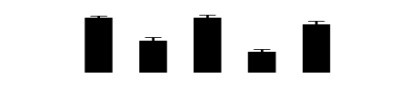 |
| TaL-8220 | Mitochondrial outer membrane porin | *Triticum aestivum* | gi|1172553 | 3 | 186 | 16% | 8.42/28944 | 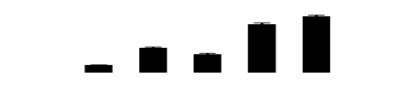 |
| TaL-8250 | Mitochondrial outer membrane porin | *Triticum aestivum* | gi|1172553 | 2 | 147 | 12% | 8.42/28944 | 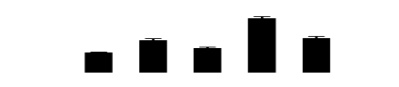 |
| **Signalling-related proteins** | | | | | | | | |
| TaL-0602 | Calreticulin | *Hordeum vulgare* | gi|439586 | 6 | 312 | 18% | 4.45/47180 | 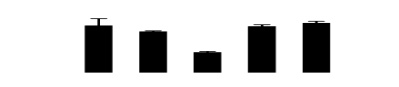 |
| **Cellular structure-related proteins** | | | | | | | | |
| TaL-2506 | Actin | *Nicotiana tabacum* | gi|461465 | 6 | 262 | 25% | 5.46/41940 | 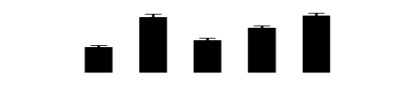 |
| **Miscellaneous** | | | | | | | | |
| TaL-6213 | S-like RNase | *Triticum aestivum* | gi|20271131 | 1 | 67 | 7% | 6.30/28320 | 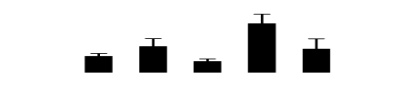 |
| TaL-5105 | Flavoprotein wrbA-like isoform 1 | *Brachypodium distachyon* | gi|357133098 | 3 | 171 | 41% | 6.21/21815 | 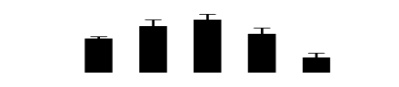 |
| TaL-8106 | NEDD8-conjugating enzyme Ubc12 | *Hordeum vulgare* | gi|326528957 | 5 | 148 | 30% | 8.35/20831 | 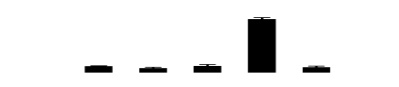 |
| **Unknown** | | | | | | | | |
| TaL-6211 | WHE3307_E04_J07ZS | *Triticum aestivum* | gi|22547245 | 2 | 70 | 19% | 8.87/23128 | 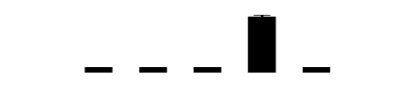 |
| TaL-1604 | Predicted protein | *Hordeum vulgare* | gi|326490439 | 5 | 210 | 13% | 5.53/51932 | 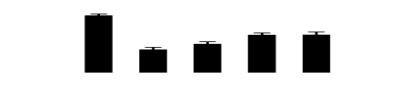 |
| TaL-7103 | Hypothetical protein | *Hordeum vulgare* | gi|2266666 | 1 | 60 | 6% | 8.56/24724 | 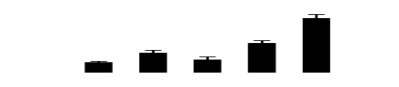 |
| TaL-7102 | Protein FLUORESCENT IN BLUE LIGHT | *Brachypodium distachyon* | gi|357157833 | 2 | 64 | 3% | 8.92/35575 | 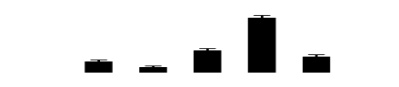 |

^a^ Spot No. corresponds to position of the spot in the gel as illustrated in Fig. 4.

^b^ Gene identification number as in GenBank.

^c^ The number of matched peptides.

^d^ The score is calculated with MASCOT. Ions score is -10*Log (P), where P is the probability that the observed match is a random event.

^e^ The sequence coverage percentage (%).

^f^ Theoretical *pI* and mass (Da) values of identified proteins estimated with MASCOT.

^g^ Time kinetics represents the average change of spot abundance at various time points 0, 18, 24, 48, R24 h (rehydration treatment). The data were taken in terms of -fold expression with respect to the control value and were log-transformed to the base two in order to level the scale of expression and to reduce the noise.
